# A robust TDP-43 knock-in mouse model of ALS

**DOI:** 10.1186/s40478-020-0881-5

**Published:** 2020-01-21

**Authors:** Shih-Ling Huang, Lien-Szu Wu, Min Lee, Chin-Wen Chang, Wei-Cheng Cheng, Yu-Sheng Fang, Yun-Ru Chen, Pei-Lin Cheng, Che-Kun James Shen

**Affiliations:** 1 Institute of Molecular Biology, Academia Sinica, Taipei, 11529 Taiwan, Republic of China; 2 Institute of Molecular Medicine, College of Medicine, National Taiwan University, Taipei, 10002 Taiwan, Republic of China; 3 Graduate Institute of Neural Regenerative Medicine, College of Medical Science and Technology, Taipei Medical University, Taipei, 11031 Taiwan, Republic of China; 4 Genomics Research Center, Academia Sinica, Taipei, 11529 Taiwan, Republic of China

**Keywords:** Amyotrophic lateral sclerosis (ALS), TAR DNA-binding protein 43 (TDP-43), Homologous knock-in mouse models with ALS-associated TDP-43 mutations, ALS-TDP pathogenesis, Autonomous spinal cord motor neuron degeneration, Mis-splicing of Bcl-2 pre-mRNA

## Abstract

Amyotrophic lateral sclerosis (ALS) is a fatal, adult-onset degenerative disorder of motor neurons. The diseased spinal cord motor neurons of more than 95% of amyotrophic lateral sclerosis (ALS) patients are characterized by the mis-metabolism of the RNA/DNA-binding protein TDP-43 (ALS-TDP), in particular, the presence of cytosolic aggregates of the protein. Most available mouse models for the basic or translational studies of ALS-TDP are based on transgenic overexpression of the TDP-43 protein. Here, we report the generation and characterization of mouse lines bearing homologous knock-in of fALS-associated mutation A315T and sALS-associated mutation N390D, respectively. Remarkably, the heterozygous TDP-43 (N390D/+) mice but not those heterozygous for the TDP-43 (A315T/+) mice develop a full spectrum of ALS-TDP-like pathologies at the molecular, cellular and behavioral levels. Comparative analysis of the mutant mice and spinal cord motor neurons (MN) derived from their embryonic stem (ES) cells demonstrates that different ALS-associated TDP-43 mutations possess critical ALS-causing capabilities and pathogenic pathways, likely modified by their genetic background and the environmental factors. Mechanistically, we identify aberrant RNA splicing of spinal cord *Bcl-2* pre-mRNA and consequent increase of a negative regulator of autophagy, Bcl-2, which correlate with and are caused by a progressive increase of TDP-43, one of the early events associated with ALS-TDP pathogenesis, in the spinal cord of TDP-43 (N390D/+) mice and spinal cord MN derived from their ES cells. The TDP-43 (N390D/+) knock-in mice appear to be an ideal rodent model for basic as well as translational studies of ALS- TDP.

## Introduction

While the genetic basis of 80% of ALS is unknown, at least 31 genes, including *SOD1* and *TARDBP* encoding the RNA/DNA-binding protein TDP-43*,* with mutations associated with ALS have been identified [13, 37]. In particular, a total of more than 50 missense mutations have been identified in the *TARDBP* gene through genetic analysis a number of familial and sporadic ALS cases [10]. Significantly, more than 95% of all ALS patients (ALS-TDP) are characterized by enhanced cleavage of TDP-43 to generate TDP-35/ TDP-25 fragments, by accumulation of ubiquitinated TDP-43/phosphorylated TDP-43, and by formation of ubiquitin(+), TDP-43(+) aggregates in the cytosol [38, 48].

TDP-43 is a ubiquitously expressed heterogeneous nuclear ribonucleoprotein (hnRNP) protein that localized primarily in the nucleus and required for multiple cellular pathways [11, 36, 38, 69] including RNA metabolism and translation. Given these ubiquitous functions, aberrant expression of TDP-43 is likely to lead to multiple pathological consequences. Indeed, depletion of TDP-43 results in early embryonic lethality in mice [40, 76], promotes cellular deficits such as the impairment of autophagy through down-regulation of Atg7 [8] and alteration of fat metabolism via suppression of Tbc1d1 [14], and causes ALS-like phenotypes in mice [74]. Furthermore, under pathologic conditions, the total amount of TDP-43 in the diseased cells is elevated [6, 34, 39, 48] in addition to its mislocalization in the cytosol and abnormal processing as mentioned above. As the pathological consequences of abnormally high levels of TDP-43, the biogenesis of many RNAs required for neural development and synaptic function are impaired [3, 53]. Mutations in or depletion of TDP-43 also affect the translation and trafficking of neuronal mRNAs [2, 16, 41, 42].

In order to study ALS-TDP disease mechanisms, different animal models have been developed which display abnormal expression of TDP-43, either a decrease [74] or increase [3, 18, 32, 40] compared to the wild type mice. The transgenic mouse models that overexpress mutant TDP-43 under different neuronal promoters exhibited MND-like phenotype or cognition deficits with neuronal loss, and the hallmarks of TDP-43 proteinopathies [48]. This included mislocalizaiton of nuclear TDP-43, abnormal post-translational modifications, and formation of insoluble ubiquitin (+) / TDP-43 (+) inclusions [36]. Unfortunately, however, overexpression of wild type TDP-43 can also cause FTLD-TDP-like [63] or ALS-like pathogenesis of the transgenic mice [45]. It is therefore difficult to assess the pathological effects of the ALS-associated mutations of TDP-43 compared to wild type TDP-43 without appropriate control of their levels of overexpression, in transgenic animals or in transfected cell culture. Notably, White et al. [72] and Fratta et al. [26] have constructed mouse models by homologous knock-in of a ALS-FTD associated TDP-43 mutation, Q331K. However, mice with either one allele or both alleles mutated only exhibit subtle ALS-FTD-like phenotypes. More recently, Ebstein et al. [24] have reported the generation of another two mouse models with knock-in of fALS-associated TDP-43 mutations, M337 V and G298S, that show relatively mild motor neuron degeneration in homozygous mice, but not in heterozygous ones, after the age of 2-year old. Here, we report the generation and characterization of two mouse models, each bearing a single ALS-TDP associated TDP-43 knock-in mutation (N390D or A315T). Remarkably, heterozygosity of the N390D mutation, but not A315T mutation, leads to a whole spectrum of male-dominant and age-dependent pathological features mimicking ALS-TDP.

## Materials and methods

### Generation of TDP-43(A315T/+) and TDP-43 (N390D/+) knock-in mice

Standard procedures were followed to generate mouse 1ine carrying different *Tardbp* (TDP-43) mutations. The targeting vector carrying mutations (A315T or N390D) on exon 6 of *Tardbp* was cloned in the BAC clone RP23-364 M1 (Invitrogen) by using the counter-selection BAC modification kit (Gene bridges). For A315T, the nucleotide G at position 943 was substituted for A; the nucleotide A at position 1168 was substituted for G for N390D. A neo-resistant gene with *loxP* sequence cassette (PGK-neo cassette) was inserted into intron 4 of *Tardbp* for ES cell screening. Two independent targeted ES cell clones (#125 and #180 for A315T, #108 and #361 for N390D) were expanded and microinjected into C57BL/6 J (The Jackson Laboratory) blastocysts to generate the chimeric mice. Knock-in ES cells carrying A315T or N390D substitution in TDP-43 were identified by standard operating procedures of the Transgenic Core Facility of Institutional Molecular Biology, Academia Sinica. To remove the PGK-neo cassette from targeted *Tardbp* allele, the germline-transmitting F1 lines were crossed with Ella-Cre mice (Tg (EIIa-cre) C5379Lmgd; The Jackson Laboratory) expressing the Cre recombinase in the whole body. The genotypes of A315T/+ or N390D/+ mice were verified by sequencing of cDNAs and genomic DNAs. All animals were maintained in a specific pathogen-free (SPF) environment under standard laboratory conditions and handled following the guidelines of the Institute Animal Care and Use Committee (IACUC) of Academia Sinica. The diseased mice were taken care of by the staff members including the feeding with soft food, spraying drinking water on the wall of cages, using soft materials for disable mice, etc.

The knock-in mice were genotyped by PCR using the forward primer 5′-GACCTCAACTGCTCTGCTTCTACC-3′ and the reverse primer 5′-AACGGAATCAA TCCTCTCCAGG-3′.

### Differentiation of mouse ESC into spinal cord motor neurons (MN) in culture

TDP-43 knock-in mice were crossed with B6.Cg-Tg (Hlxb9-GFP)1Tmj/J (Hb9:GFP; The Jackson Laboratory) transgenic mice to obtain offspring of the genotypes of TDP-43 (A315T/+); Hb9:GFP and TDP-43 (N390D/+); Hb9:GFP, respectively. The ESCs from 3.5 day embryo were cultured and differentiated into spinal MN as depicted in Fig. 4 following the protocols described by [12, 73]. Briefly, in inductive phase, ESCs were cultured in differentiation medium (45% Advanced DMEM/F12 (Gibico), 45% Neurobasal (Gibico), 10%l Knockout-SR (Gibico), 2 mM L-glutamine (Millipore)) to form the embryonic bodies (EBs) on day1. On day 2, EBs were added with RA (Retinoic acid, Sigma-Aldrich) and SAG (Smoothened agonist, Cayman Chemical), a Shh pathway activator [44] and cultured for another 2 to 3 days to promote MN differentiation. On day 5, cells expressing GFP under the control of spinal cord motor neuron-specific promoter Hb9, i.e. MN, would appear, and the EBs were dissociated with 0.25% Trypsin-EDTA (Gibco), plated on coverslips pre-coated with 0.01% poly-D-lysine (Sigma)/ 0.01% ornithine (Invitrogen), 5 μg/ml laminin (Invitrogen), and cultured in the MN medium (45% Advanced DMEM/F12, 45% Neurobasal, 10%l Knockout-SR, 2 mM L-glutamine, 1x B27 (Gibico), 1x N2 supplement (Gibico), 10 ng/ml GDNF (Peprotech). When required, GFP (+) MNs were purified by flow-sorting in FACSAriaII SORP.

### Cell lines

Neuro 2a (N2a) cells were cultured in minimum essential medium (MEM, Gibico) supplemented with 10% FBS (Gibico), 1% sodium pyruvate (Invitrogen), 10% FBS (Gibico) and 1% antibiotics (100 IU/mL penicillin and 100 g/mL streptomycin, Invitrogen), whereas HEK293T cells was cultured in Dulbecco’s modified Eagle’s medium (DMEM, Gibico) supplemented with 10% FBS and 1% antibiotics.

### Behavior tests

#### Accelerating rotarod

Mice were trained for 3 days and tested following the procedures described in Mandillo et al. (2008). In brief, the mice were placed on a rod (Ugo Basile Rota-Rod 47,600) rotating at 4 rpm constant speed. In testing phase, the rotation speed was accelerated from 4 to 40 rpm in 5 min. Latency and fall-off rpm of each mouse was recorded when the mice fell from the rod.

#### Hindlimb-clasping test

The test was carried out as described previously [30]. The mice were suspended by grasping their tails and their hindlimbs position were observed for 10 s. The normal mice consistently kept their hindlimbs away from the abdomen. Hindlimbs of the knock-in mice having motor dysfunction would be retracted toward or touching the abdomen during the suspended time.

#### T-maze test

T-maze apparatus was used to measure spatial working memory following the procedures described by Deacon and Rawlins (2006) [21] and Shoji et al., (2012) [59]. In brief, the mice were in food restriction (3 g ± 0.5 g/mouse/day) and treated with the reward pellets (40 mg/mouse/day) for 3–4 days before the test. They then stayed in the maze for habituation for at least 5 min and repeat 5 times until they were accustomed to reward pellets before the test. Mice were tested on 2 trials in this test including sample phase (learning and memory phase) and choice phase (reversal learning phase) as shown in Additional file 2: Figure S2a. Each trial is repeated five times a day and continued until the mice can get over 80% correction for 3-month old +/+ mice, and over 60% correction for the 24-month old +/+ mice. The standby site of young mice is 30–35 cm from the T intersection; the old N390D/+ mice were 10 cm from the T intersection because they would have movement problem. Between each run, the apparatus was cleaned with 75% alcohol to remove the effect of olfactory.

### Examination of hindlimb skeletal muscles and soleus

**Fig. 1:**
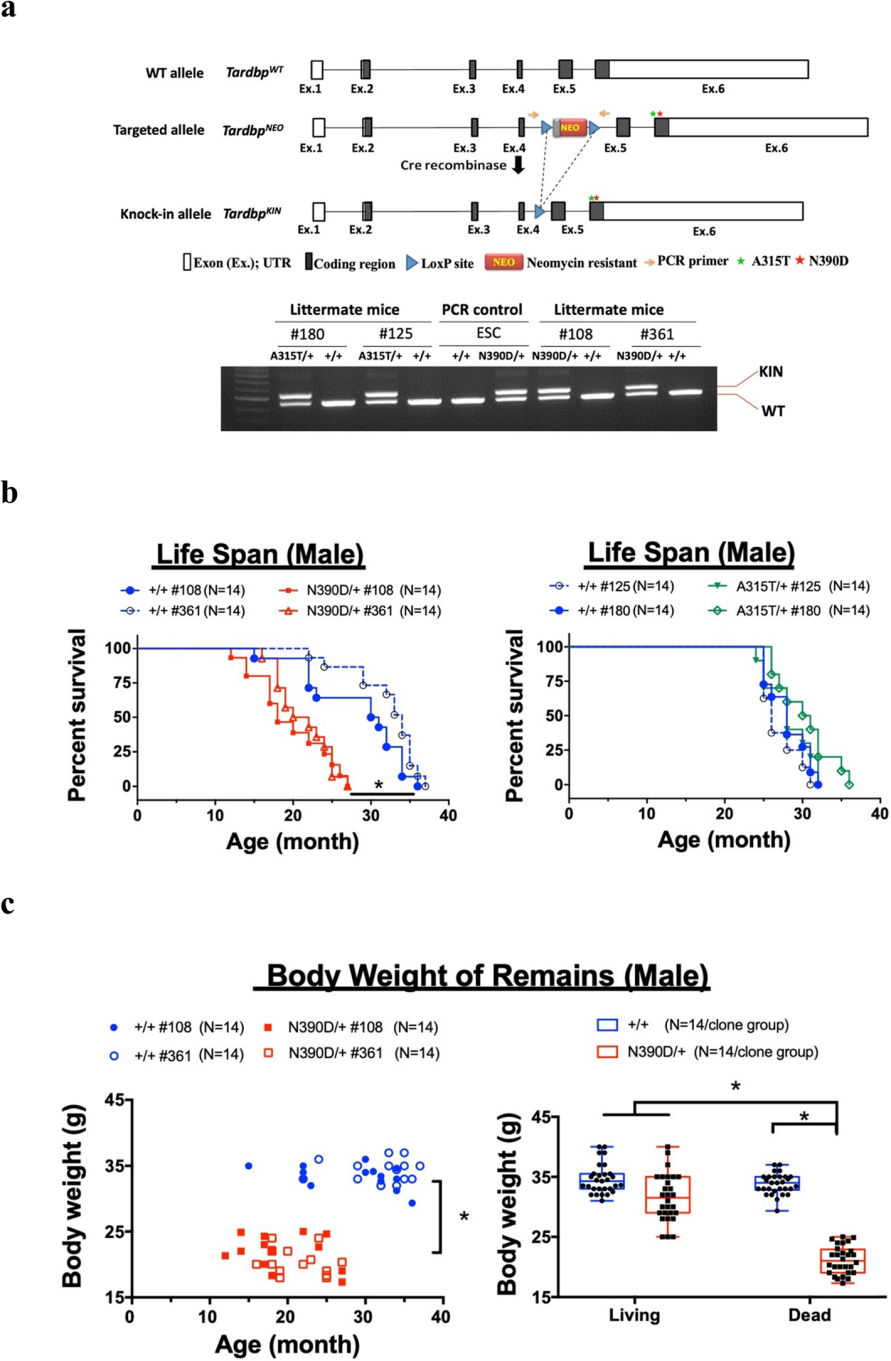
Age-dependent ALS-like phenotypes of Heterozygous male N390D/+mice. **a** Schematic diagram of targeted knock-in (KIN) of ALS-associated mutant TDP-43, A315T (green star) and N390D (red star), in mouse *Tardbp* gene. Both sides of the NEO cassette in the targeted allele (*Tardbp*^*NEO*^) was flanked by *lox P* sequence (arrowhead). The genotypes of mice carrying the different knock-in allele (*Tardbp*^*KIN*^) were validated by PCR, as exemplified for two heterozygous lines each for N390D and A315T. #125 plus #180 and #108 plus #361 are the name of mouse lines of A315T and N390D, respectively. For more details, see Methods. **b** The survival rates of two lines each of A315T/+ (right panel) and N390D/+ male mice (left panel) are compared to their +/+ littermates. Note the significantly shorter life span of N390D/+ male mice, but not A315T/+ male mice, than the +/+ male mice. *p < 0.05. **c** Comparison analysis of the average body weight of the remains of N390D/+ male mice to that of the living mice weighed before they died. The left panel shows the comparison of the weight of remains between +/+ and N390D/+ male mice in a manner consistent with the survival curve. The right panel shows the comparison of the body weight of the remains of N390D/+ male mice to the body weight recorded when they are still alive. * *p* < 0.05. The numbers (N) of mice analyzed per group are listed in the figure

The tricep surae consisting of a pair of gastrocnemius muscle and one soleus muscle is the major muscle of the calf of human and most of mammals. After cutting down the right hind limbs, the diameters of the fresh calf circumference were measured. The standard position of the calf muscle for measurement was the widest circumference of the calf as shown in the carton of Fig. 1d, approximately 1.2 cm from the calcaneum and parallel to the tibia. The soleus is a powerful muscle involved in standing and movement. After isolation from the left calf muscles, the soleus was weighted by electronic micro balance. The right calf and left soleus were also immersed in 4% PFA overnight (post-fixation) for further analysis.

### NMJ staining

**Fig. 2:**
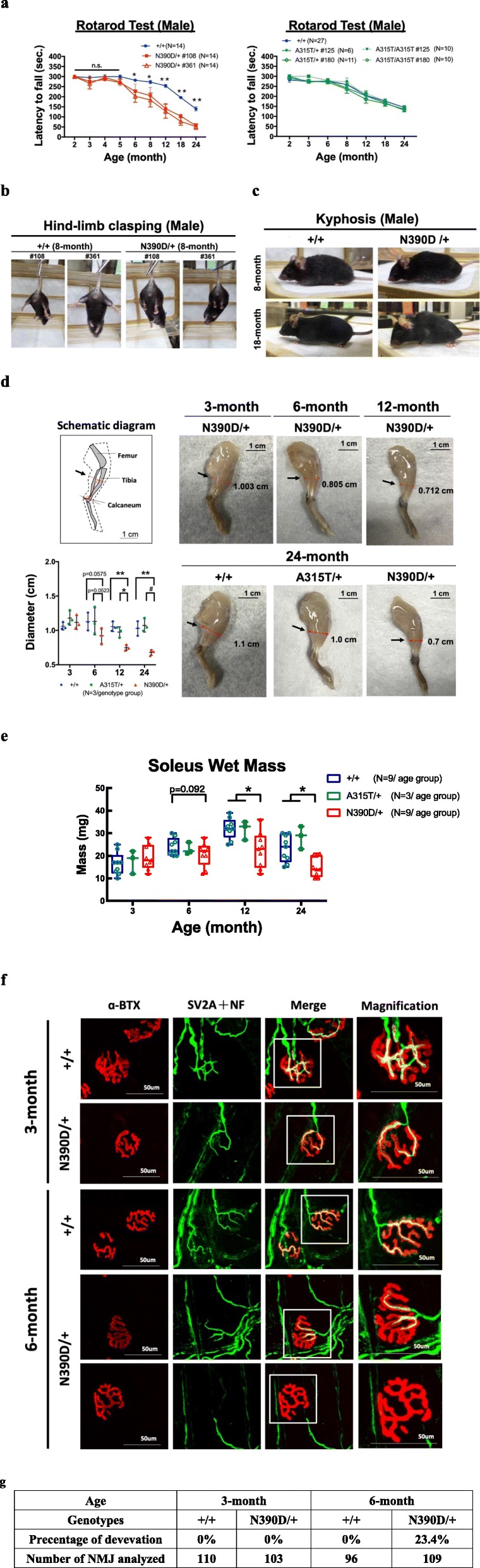
Age-dependent motor dysfunction and muscle atrophy of heterozygous male N390D/+ mice. **a** Rotarod test of heterozygous knock-in mouse lines. Both lines of N390D/+ male mice (left panel) exhibited motor dysfunction at the age of 6 months, but not the two A315T/+ mouse lines (right panel). The average time periods of mice staying on the rotarod (mean ± SEM) at different ages are shown in the line graphs. *p < 0.05; **p < 0.01, n.s., not significant. The numbers (N) of mice analyzed per group are listed in the figure. **b** Hind-limb clasping test. Exemplified are the abnormal clasping behavior of 8-month old N390D/+ male mice (right 2 panels) and their +/+ littermates (left 2 panels). **c** ALS-like kyphosis phenotype of N390D/+ male mice (right panels) and the +/+ littermates (left panels) at the ages of 8 months and 18 months, respectively. **d** Cartoon illustration of the standard measurement position (arrow) of the calf muscle by red I symbol. Photos of the hind-limb muscles of 3-, 6-, 12- and 24-month old N390D/+ male mice, and 24-month old +/+ and A315T/+ male mice, respectively. The statistical results are shown on the lower left side. The red lines point to the measured sites of the muscle. Scale bars 1 cm. *p < 0.05; **p < 0.01; #*p* < 0.001. **e** The soleus muscles of hind-limb were isolated from +/+, A315T/+ and N390D/+ male mice at different ages. *p < 0.05. **f** The NMJ of soleus from +/+ and N390D/+ male mice were analyzed by whole-mount IF staining using α-Bungarotoxin-555 (α-BTX, red) and anti-SV2A (green, a synaptic vesicle marker) plus anti-NF (green, a nerve marker). The white box indicates the magnified region. N = 3 (randomly chosen from each of the two independent lines) per group. Scales bars 50 μm. **g** The percentages of denervation of NMJs of the soleus muscles of hind-limb at the ages of 3 and 6 months, respectively. The numbers of NMJ analyzed are list in the bottom of the table

The NMJ staining was carried out as the methods described by Tremblay et al., [62]. The fixed soleus muscle samples were teased apart to fibers by using forceps, and the soleus muscle fibers were permeabilized with 10% FBS, 1% Triton-X100 diluted in PBS for 1 h at room temperature. The axons and nerve terminals of NMJ were labeled with antibodies including anti-SV2A (1:200 dilution; Cell Signaling) and anti-NF (1:200 dilution; Proteintech) for 72 h at 4 °C, incubated with the secondary antibody Alexa Fluor 488 for 48 h at room temperature, and then incubated with Alexa Fluor 594-conjugated α-Bungarotoxin (α-BTX; 2.0 μg/ml; Thermo Fisher Scientific) for 2 h at room temperature. The images of MNJ were acquihired with Z-stacking and projected into 2D images from use of Zeiss LSM 780 Confocal Microscope. The percentage of denervation of the NMJ mentioned in the text was calculated in the formula $\%=\frac{\mathrm{the}\ \mathrm{number}\ \mathrm{of}\ \mathrm{endplates}\ \mathrm{withcpmplete}\ \mathrm{loss}\ \mathrm{of}\ \mathrm{SV}2\mathrm{A}\ \mathrm{and}\ \mathrm{NF}\ \mathrm{s}\mathrm{ignal}\mathrm{s}}{\mathrm{total}\ \mathrm{number}\ \mathrm{of}\ \mathrm{endpaltes}\kern0.5em \mathrm{with}\ \upalpha -\mathrm{BTX}\ \mathrm{s}\mathrm{ignal}\ }$. The numbers of soleus NMJ examined in the 3-month old and 6-month old +/+ and N390D/+ male mice are indicated in Fig. 2g.

### Proteasome chymotrypsin-like activity assay

The forebrain and spinal cord proteins were extracted freshly. This assay was carried out in 96 well plates with 50 μg of the extracts lysate and fluorescently labeled substrate succinyl-Leu-Leu-Val-Tyr-7-amido-4-methylcoumarin (Suc-LLVY-AMC; final conc. Was 100 μM; BML-P802–0005, Enzo Life Sciences, NY) in assay buffer [20 mM Tris-HCl (pH = 7.4), 5 mM MgCl2, 5 mM DTT and 2 mM ATP]. The kinetics of the Suc-LLVY-AMC cleavage was measured spectrofluorometrically at 5 min intervals for 1 h under 37 °C by using an EnSpire Multiplate Reader (Ex/Em = 360 nm/460 nm; PerkinElmer). Negative control assay was conducted in the presence of the specific proteasomal inhibitors MG-132 (474,790, Calbiochem) and Lactacystin (70,988, Cayman).

### Immunofluorescence staining analysis

For staining of the brain and spinal cord, 14 μm thick brain sections and 10 μm thick spinal cord sections were prepared as described before [74]. Antibodies for IF staining of these sections included goat anti-choline acetyltransferase (ChAT; 1:800 dilution; Millipore), mouse anti-NeuN (1:200 dilution; Millipore), rabbit anti-TDP-43 (1:1000 dilution; Proteintech), rabbit anti-pTDP-43 (1:500 dilution; Proteintech), mouse anti-ubiquitin (1:200 dilution; Novus), and rat anti-GFAP (1:200 dilution; Invitrogen).

For staining of ESC-derived MN, the culture medium was removed and the cells were washed gently with PBS. MN on coverslips were fixed by freshly made and pre-colded 4% paraformaldehyde for 20 min. The samples of spinal cord sections or ESC-derived MN were then permeabilized with PBS/0.5% Triton X-100 for 7 min at room temperature. After blocking with 2% fetal bovine serum (FBS) for 1 h at room temperature, the samples were incubated overnight at 4 °C with one or more of different antibodies, including goat anti-choline acetyltransferase (ChAT; Millipore), rabbit anti-TDP-43 (Proteintech), and mouse anti-Tau (1:200 dilution; Thermo Pierce). After washing, the samples were incubated with DAPI (Invitrogen) plus Alexa Fluor 488-conjugated, Alexa Fluor 546-conjugated, and Alexa Fluor 647-conjugated secondary antibody (Invitrogen) for 1 h at room temperature. The images were analyzed on a Zeiss LSM 780 confocal microscope.

### Western blotting analysis

The mouse tissues (200 mg /ml) were extracted with RIPA buffer (0.1% SDS, 1% Nonidet P-40, 0.5% sodium deoxycholate, 5 mM EDTA, 150 mM NaCl, 50 mM Tris-HCl, pH 8.0) or urea buffer (7 M urea, 2 M thiourea, 4% CHAPS, 30 mM Tris-HCl, pH 8.5) containing protease and phosphatase inhibitors (Roche). After homogenization and centrifugation of the tissue at 13,000 rpm 4 °C for 30 min, the solution from the urea buffer-derived extract was defined as the “total protein extract”, and the supernatant from the RIPA buffer extract(s) was defined as the “soluble fraction”. The pellet from the RIPA buffer-derived extract was washed by RIPA buffer for 3 times, dissolved in urea buffer, and defined as the “insoluble fraction”. The cellular extracts of cultured MN were prepared in the following way. The cultured spinal MN (see below) were purified by GFP (+)-based sorting in FACSAriaII SORP. The purified GFP (+) MNs were cultures for different days and lysed with lysis buffer. 1*10^6^ MNs were lysed with either RIPA buffer or urea buffer. For in vitro phosphorylation of TDP-43 as a positive control of Western blotting, the recombinant TDP-43 was expressed in *E. coli*. and purified through Nickel affinity column. The purified TDP-43 was then prepared in 3 μM in 10 mM Tris, pH. 8.0, 5 mM MgCl2, 200 μM ATP, and 1 μl Casein Kinase 1 (New England BioLabs) in 100 μl solution. The reaction was incubated at 30 °C for 2 h and stopped by adding SDS containing sample loading dye.

The different protein extracts were separated by 12–15% SDS-PAGE and immunoblotted with the appropriate primary antibodies (anti-TDP-43 and anti-pTDP-43 from Proteintech, anti-ubiquitin from Proteintech, anti-histon H4 from Millipore, anti-LC3 from NOVUS, anti-p62 from Proteintech, anti-Bcl-2 from Proteintech, and anti-tubulin as well as anti-actin from Sigma) and then the secondary antibodies. The bound antibodies were detected by using the chemiluminescence Western blotting detection reagent ECL (Amersham Pharmacia Biotech, Piscataway, NJ). The expression levels of different proteins were compared by measuring their band intensities on the blots with Image J software (NIH).

### Preparation of nuclear and cytosolic extracts

The standard process followed that by Arnold et al. (2013). Briefly, spinal cord was lysed gently with 10x (vol./weight) hypotonic buffer A (10 mM Hepes-KOH, pH 7.4, 10 mM KCl, 1.5 mM MgCl, 0.5 mM EDTA, 0.5 mM EGTA) containing protease inhibitors (Roche) by homogenization. After 15 min on ice, 0.5% NP-40 was added and the samples were vortexed and centrifuged at 800 g for 5 min at 4 °C. The supernatant was defined as the cytosolic fraction. For preparation of the nuclear extract, the nuclear pellet was washed with hypotonic buffer A, added 5x (vol/wt) extraction buffer (10 mM Hepes-KOH, pH 7.4, 0.42 M NaCl, 2.5% (vol/vol) glycerol, 1.5 mM MgCl, 0.5 mM EDTA, 0.5 mM EGTA, 1 mM DTT) containing protease inhibitors, and then incubated at 4 °C while rotating at 60 rpm for 40 min.

### Survival rate analysis of cultured MN

A modified MN induction protocol was used for this analysis. On day 5 of the differentiation phase, the EBs were directly cultured in the MN medium without dissociation by trypsin. The EBs were then examined by FACS (LSRII-12P) for 14 days in culture. The percentage of the GFP (+) MN in the EBs on the 1st day was defined as 100% and the folds of change on the following days were quantitated and compared for the WT and mutant MN.

### RT-PCR and RT-qPCR analysis

Total RNAs from the tissues or cells were isolated following the standard protocol using Trizol reagent (Thermo Fisher Scientific). cDNA synthesis was carried out using SuperScript II reverse transcriptase (Invitrogen) and subjected to PCR. Alternatively, real-time PCR (qPCR) using SYBR Green PCR Master Mix (Applied Biosystems) and ABI 7500 real-time System was carried out. All data were analyzed after normalization to the expression level of the *Gapdh* gene. The sequences of the PCR primers are listed below:

*Tardbp* forward primer: 5′- GGTAATCCAGGTGCTTTG-3′

*Tardbp* reversed primer: 5′- CCTGCATTTGATGCTGACCC-3′

*Bcl-2* splicing forward primer (P1):5′-TTCGGGGAAGGATGGCGCAAGC- 3′

*Bcl-2-201* forward primer (P2): 5′- ACGGAGGCTGGGATGCCTTTGTGG-3′

*Bcl-2* reversed primer (P3): 5′- TCACTTGTGGCCCAGGTATGC-3′

*Eif4h* splicing forward primer: AAGCTAGCTATCCATGGCGGACTTCGACACCTACGACG-3′

*Eif4h* splicing reversed primer: 5′-CCCGGGAGCTCTCATTCTTGCTCCTTTTGAACGAC-3′

*Mapt* forward primer: 5′-AGGAAATGACGAGAAGAAAGC-3′

*Mapt* reversed primer: 5′-GACCCTGGAGGAGTCTTAGG − 3′

*Rbm39* forward primer: 5′- GGCCGCTACAGAAGTCCTTACTCC - 3′

*Rbm39* reversed primer: 5′-ACCCAATCTTTCCTCGGATGG- 3′

*ANkrd12* forward primer: 5′- TTACTATTAGCCCATCAAGAAATGAAG − 3′

*ANkrd12* reversed primer: 5′- TATGTCCTGGATCTGAATCTGTGTC-3′

### Expression plasmid construction and DNA transfection

cDNAs of mouse wild type TDP-43, TDP-43 (A315T) and TDP-43 (N390D) with addition of a Myc epitope tag to the 3′ -end were generated by RT-PCR of different mouse spinal cord RNAs as the templates. The PCR primers used were: forward 5′-CCG CTC GAG CGG ATG TCT GAA TAT ATT CGG GTA AC-3′; reverse 5′- TGC TCT AGA GCA CAT TCC CCA GCC AGA AGA C-3′. These cDNA fragments were first cloned into pGEM-T vector (Promega Corporation) and then subcloned into the XhoI/ XbaI sites of pEF-myc vector (Promega Corporation). The generation of expression plasmids carrying human wild-type TDP-43, TDP-43 (A315T), and TDP-43 (N390D) cDNA, respectively, was described in Wu et al. [75].

### DNA transfection

The N2a cells were transfected with the pEF-myc vector or different mouse TDP-43 expression plasmids. HEK293T cells were transfected with the vector or different human TDP-43 expression plasmids [75]. DNA transfection was carried out with Lipofectamine 2000 (Invitrogen) according to the manufacturer’s protocol. The amount of the plasmid DNA used in each transfection was 4 μg per 6-cm dish. The cells were harvested at 24 h post-transfection and analyzed by different assays, e.g. Western blotting, calcium imaging, etc.

### Cyclohexamide chase assay

The procedures followed those by Huang et al. (2014) In brief, MNs on day 2 were treated with cycloheximide (20 mg/ml) for different time periods (2, 4, 8, 12 and 24 h). The levels of TDP-43 in the treated MN were analyzed by Western blotting and the relative intensities of the TDP-43 bands were quantified by AlphaEaseFC software.

### Calcium imaging

MNs were dissociated on differentiation day 5, plated on coated 22 X 22 mm glass coverslips, and grown in MN medium until calcium imaging experiments. HEK293T cells were cultured on 0.1% gelatin-coated 22 X 22 mm glass coverslips and transfected with plasmid DNAs for 24 h before calcium imaging. Before calcium imaging, the MNs were treated with 2uM Fura-2-AM (Invitrogen) in HBSS (Invitrogen) containing 2 mM CaCl_2_ in the dark chamber and incubated at 37 °C, 5% CO_2_ for 45 min. The excess Fura-2-AM was washed out with HBSS (without CaCl_2_) and incubated for an additional 30 min in HBSS (containing 2 mM CaCl_2_) for recovering, and then the coverslips were transferred onto the recording chamber of an inverted fluorescence microscope (Zeiss Axiovert 200) equipped with a 20 X objective lens and MetaFluor (Molecular Devices) acquisition and analysis software. The fluorescence signals at 510 nm were acquired every 2 s in 5 min by UV excitation at wavelengths of 340 nm (indicating calcium ion-bound- Fura-2-AM) and 380 nm (indicating calcium ion-free-Fura-2-AM), respectively. The formula $R=\frac{\Delta 340}{\Delta 380}$, in which the ∆ indicated the values of 340 nm or 380 nm minus their background values, was used to calculate and compare the relative levels of intracellular calcium ion of different types of MN in culture.

### Statistical analysis

Statistical differences were analyzed by Kaplan–Meier and log rank test(s) for the survival rates, two-way ANOVA and Bonferroni post hoc analysis for multiple group comparisons, and the unpaired Student’s t-test for two group comparisons (SPSS version 15.0, SPSS Inc. and Prism (version 7, GraphPad software). The error bars of mouse behavior were showed by SEM, and the error bars of molecule and cellular analysis were displayed by SD.

### Data collection

The images of immunofluorescence staining were acquired with Z-stacked on a Zeiss LSM 510 META confocal microscope software; basically, one slide contain 3 spinal cord sections or 2 brain sections of one mouse at least; each sample of IF staining will duplicate; the merge images and analysis such as motor neuron number and axonal length were measured/calculated via ImageJ (NIH). The bands intensities of RT-PCR and Western blotting were also quantified through ImageJ or AlphaEaseFC 4.0 (Alpha Innotech) or ImageJ (NIH). The raw data of Calcium imaging is recorded via MetaFluor (Molecular Devices) acquisition and analysis software, and the result were calculated using Prism 7.

## Results

### An ALS-like phenotype of mice bearing a single TDP-43 N390D mutation, but not an A315T mutation

We generated mouse lines bearing homologous knock-in of human ALS-associated base substitutions, A315T and N390D, respectively. The TDP-43 A315T was identified in all affected members but not the healthy control subjects of several European families [27, 33], while N390D was identified in a sporadic ALS-TDP patient from Quebec [33]. We substituted the conserved nucleotide G at position 943 with A (for A315T) or A at position 1168 with G (for N390D) in the mouse *tardbp* gene (Fig. 1a). Although the ALS-TDP patients carried A315T or N390D mutation on only one allele [27, 33], we did try to obtain homozygous offspring for analysis by intercrossing the heterozygous knock-in mice. Either A315T/+ or N390D/+ mice were fertile and the male mice have normal courtship behavior. However, in contrast to homozygous A315T/A315T mice, no homozygous N390D/N390D mice could survive to the age of 3 weeks. In parallel, most of the dead newborn pups were genotyped as N390D/N390D and the rest were N390D/+ (see Additional file 1: Figure S1a). Notwithstanding, the amount of TDP-43 protein in the spinal cord of homozygous N390D/N390D mice was significantly higher than the wildtype mice (Additional file 1: Figure S1c). Therefore, the death cause of N390D/N390D pups could result from a lethal effect of the homozygous N390D/N390D mutation on early development of the mice, as also observed for the homozygous human TDP-43 A315T knock-in mouse model generated by Stribl’s group [61]. In addition, the male-to-female ratio of the survived N390D/+ pups was 1 to 2.6 approximately (Additional file 1: Figure S1b). Notably, the disease-onset ages of individual N390D/+ female mice were highly variable as reflected by the rotarod test, and the survival time of N390D/+ female mice was significantly longer than males (Additional file 1: Figure S1d), with average age being 25.5 ± 6 months. Around 30% of the N390D/+ female mice were indistinguishable from the +/+ female mice suggesting that estrogen might exert a protective effect [20, 47, 50]. Based on the above, we concentrated on the use of the N390D/+ male mice for all analysis depicted in the following. Although the cause(s) for their eventual death was unknown, the N390D/+ male mice derived from 2 independent ESC lines (#108 and #361) displayed a shorter life span than either +/+ or A315T/+ male mice, with average age of 19.5 ± 2 months (Fig. 1b). Like the heterozygous Q331K/+ mice studied by White et al., [72] the N390D/+ male mice from either line had similar body weight as the +/+ male mice (Additional file 1: Figure S1e) although conspicuous weight loss was observed in their remains (Fig. 1c), possibly in part resulted from the lack of nutrition at the end stage of their lives. These data indicate that loss of body weight was not the initial pathological evens of ALS in N390D/+ male mice.

Rotarod and T-maze tests were used to assess whether the knock-in mice would develop ALS- and/or FTLD-like phenotypes. Surprisingly, while heterozygous N390D/+ male mice from either line #108 or #361 exhibited significant motor dysfunction at the ages of 6 months and older, heterozygous and homozygous A315T male mice appeared indistinguishable from +/+ littermates (Fig. 2a). Notwithstanding, the young as well as old N390D/+ male mice did not develop impaired performance in the T-maze task, as shown in Additional file 2: Figure S2a. Furthermore, there was no loss of neurons in the dentate gyrus (DG), CA1 and CA3 regions of hippocampus of N390D/+ male mice in comparison to the +/+ male mice (see Additional file 2: Figure S2b**)**. Over time, the N390D/+ male mice from either line #108 or #361 started to show abnormal hind limb-clasping and kyphosis at around 8 months of age, as exemplified in Fig. 2b and c, and displayed spastic and trembled gait at 18 months and beyond (Additional file 9: Movie S1 and Additional file 10: Movie S2), comparing to age-matched +/+ mice (Additional file 11: Movie S3). Since the paralysis happened in the hindlimb of N390D/+ mice, we guessed that the hindlimbs were affected earlier than the forelimbs. Also, we analyzed mainly the soleus muscle, which was one of the main muscles that control walking, running and jumping, following the study by Tremblay et al. [62] on the denervation of neuromuscular junctions in SOD-1 mice. In contrast to young N390D/+ male mice, the 12-month and 24-month old N390D/+ male mice exhibited significant skeletal muscle atrophy when compared to +/+ or A315T/+ mice, as represented by decrease of the wet weight of soleus muscle and reduction of the calf muscle volume (Fig. 2d and e). Wet mass was a relatively simple and preparatory sign for muscle atrophy as also used by others, eg. Goto et al., [28], Dang et al., [19], etc. Furthermore, while the innervation of neuromuscular junctions (NMJ) in the soleus muscle of 3-month old N390D/+ mice appeared to be normal as the +/+ mice (yellow color, Fig. 2f), denervation of the NMJ of soleus muscle was detected in N390D/+ mice at the age of 6 months, as exemplified in the representative images of the bottom panel in Fig. 2f, by approximately 23% in comparison to the +/+ male mice (Fig. 2g). The extent of NMJ denervation progressively increased with further aging of the N390D/+ mice (data not shown). Based on the similarities of N390D/+ male mice derived from lines #108 and #361 with respect to their survival curves, body weight changes and rotarod test result, we mixed used mice from the two lines for the following molecular and cellular pathology analysis. The same strategy was applied to the analysis of A315T/+ and +/+ mice.

### Molecular and cellular pathology of the TDP-43 (N390D/+) male mice

#### Accumulation, enhanced cleavage, elevated phosphorylation and increased insolubility of spinal cord TDP-43

**Fig. 3:**
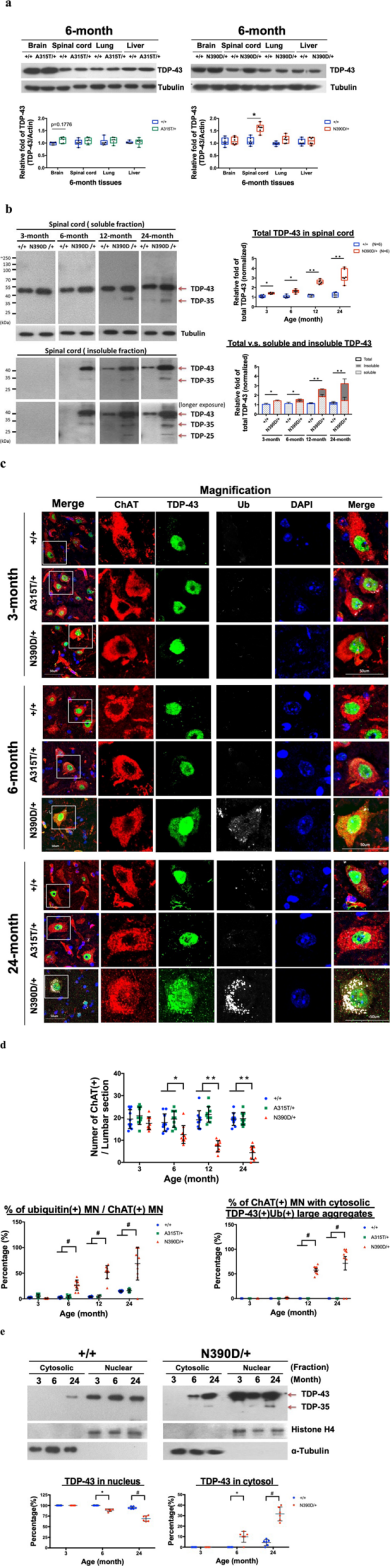
Patho-signature analysis of TDP-43 in spinal cord of N390D/+ knock-in male mice. **a** Expression of TDP-43 in different tissues of 6-month old A315T/+ (left panel) and N390D/+ male mice (right panel). Statistical analysis is shown in the box plots (min to max with all points). **b** Expression of TDP-43 (including TDP-35 and TDP-25) in the soluble and insoluble fractions of spinal cord. The relative fold of total spinal cord TDP-43 is exemplified in the right upper box plots (with all points). The lower stacked bar plot (mean ± SD) indicates the proportions of soluble and insoluble TDP-43 in the spinal cord of +/+ and N390D/+ male mice. **c** Immunofluorescence co-staining of spinal cord lumbar sections from +/+ and N390D/+ male mice using anti-TDP-43 (green), anti-ubiquitin (Ub, gray) and anti-ChAT (red; a motor neuron marker). DAPI (blue) indicates the locations of the nuclei. Image of the motor neurons are magnified from the ventral horn of the spinal cord. The vessel-like signals are likely due to staining of axons of the motor neurons. The white line boxes in the first column indicate the magnified regions of panels on the right. The scale bars are 50 μm. **d** Statistical comparison of the ChAT (+) motor neuron numbers per section, the % of ubiquitin (+) MN among the ChAT (+) MN, and the % of ChAT (+) MN with large ubiquitin (+) TDP-43 (+) MN aggregates. **e** The cytosolic and nuclear distribution of spinal cord TDP-43 in the N390D/+ and +/+ male mice at different ages. Histone H4 (nuclear marker) and ɑ-tubulin (cytosolic marker) were used to validate the fractionation of cellular extracts. The scatter plot (mean ± SD) deduced from the Western blotting data are shown below the blots. *N* = 6 (3 mice from each of the two independent lines) per group in **a**, **b** and **e**. N≧3 (randomly chosen from each of the two independent lines) per group in **c** and **d**. Significantly different represented in **a**-**e**: *p < 0.05, **p < 0.01, #*p* < 0.001

**Fig. 4:**
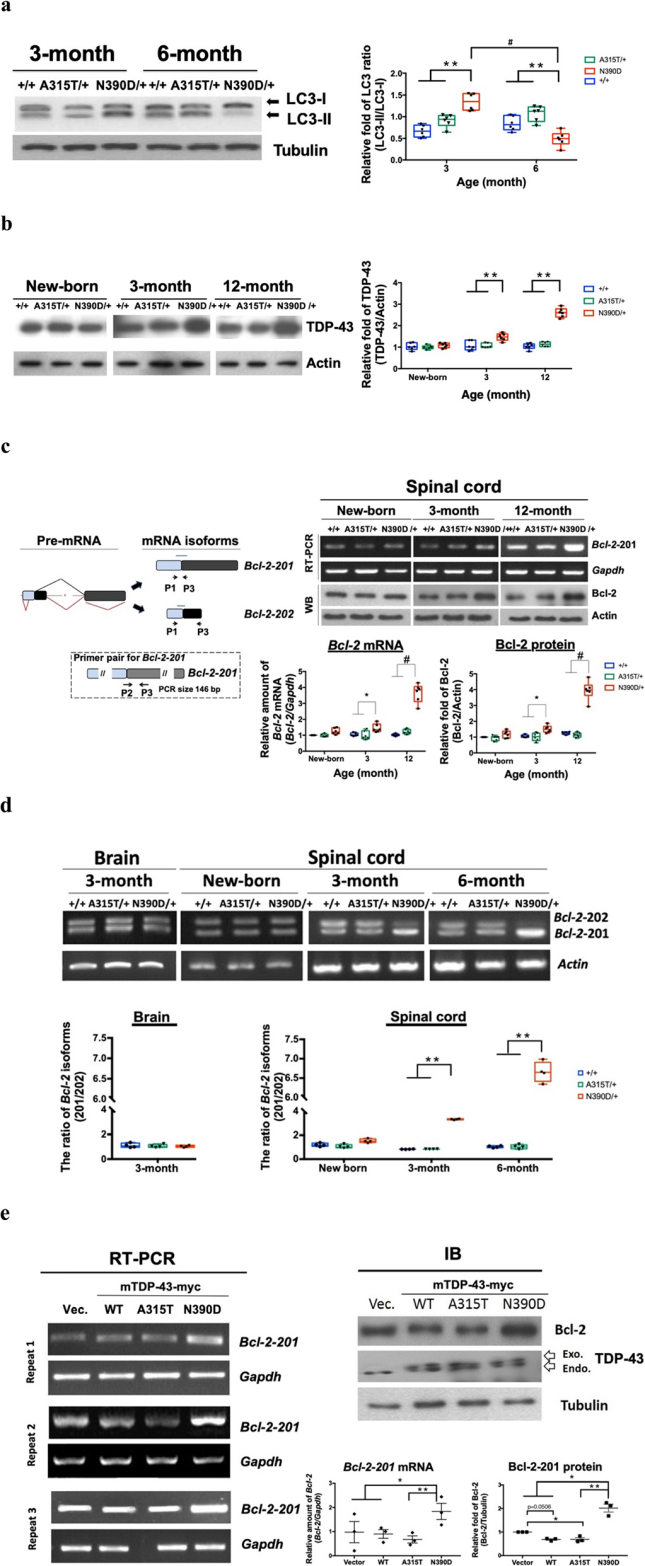
Effects of TDP-43 (N390D) mutation on autophagy and Bcl-2 expression. **a-b** Expression of LC3-I and LC3-II (**a**) and TDP-43 protein (**b**) in the spinal cord of +/+, A315T/+ and N390D/+ male mice at different ages. The blots are exemplified on the left, and the statistical analyses are shown on the right box plot (min to max with all points). **c** The left scheme is the alternative splicing of *Bcl-2* pre-mRNA to generate the *Bcl-2-*201 mRNA (the black lines) and *Bcl-2-*202 mRNA (the red lines). The 3 arrows (P1, P2 and P3) indicate the primers used for RT-PCR. Blue lines indicate location of the coding region. The boxes with different colors are the different exons. The arrows in the dashed frame are the primers (P2, P3) used for detecting the *Bcl-2*-201 mRNA. The expression of *Bcl-2-*201 mRNA and Bcl-2 protein in the spinal cords of A315T/+, N390D/+ and +/+ male mice at different ages are exemplified in the upper right two panels, with the statistical analysis of the levels of *Bcl-2* mRNA and Bcl-2 protein shown in the lower right two box plots (min to max with all points). **d** The changes of the relative ratio of the two *Bcl-2* mRNA isoforms. *N* = 4 (2 from each of the two independent lines) per group. **e** Comparison of the levels of Bcl-2 expression in transfected N2a cells. The levels of *Bcl-2*-201 mRNA and Bcl-2 protein were assayed by RT-PCR (left panels) and by Western blotting (right upper panel), respectively. The statistical analysis of the levels of *Bcl-2* mRNA and Bcl-2 protein are in the right lower 2 dot plots (mean ± SD). *N* = 6 (3 from each of the two independent lines) per group in **a** and **c.** Significantly different represented in **a**-**e**: *p < 0.05, **p < 0.01, #*p* < 0.001

Changes in a range of molecular and cellular characteristics were associated with the ALS-like phenotypes of the N390D/+ male mice described above. First, in contrast to A315T/+ male mice, the expression level of TDP-43 protein was elevated in the spinal cord, but not in other tissues, of N390D/+ male mice, and progressively elevated with age in the spinal cord, e.g. newborn, 3, 6, 12 and 24 months (Fig. 3a and b, Fig. 4b and Additional file 4: Figure S4a), despite the similar levels of *tardbp m*RNA (see Additional file 3: Figure S3, a and b). Second, there was an age-dependent enhancement of cleavage of TDP-43 to generate 35-kDa and 25-kDa fragments in the spinal cord of post-symptomatic N390D/+ mice (Fig. 3b), which was characteristic of the spinal cord of ALS-TDP patients [49, 56]. The enhanced cleavage of TDP-43 was accompanied by significantly increased fraction of insoluble TDP-43/ TDP-35/ TDP-25 in the cellular extracts from mouse spinal cords of N390D/+ male mice at the age of 6 months and beyond (Fig. 3b and Additional file 3: Figure S3c). Thirdly, the amount of the phosphorylated TDP-43 (pTDP-43), which was identified as a component of the ubiquitin-positive inclusions (UPIs) in diseased neurons of ALS and FTLD [48], was increased significantly in the spinal cord (see Additional file 4: Figure S4a), but not the forebrain (see Additional file 4: Figure S4b), of mice at the age of 12 months and beyond.

#### Age-dependent mislocalization of TDP-43 and pTDP-43, accumulation of ubiquitinated proteins in spinal cord MN, and loss of spinal cord MN

A characteristic of ALS pathogenesis is the formation of large cytosolic TDP-43(+), ubiquitin(+) aggregates, and depletion of nuclear TDP-43 in diseased motor neurons [48]. The pathogenic significance of these aggregates with regard to the initiation and/or progression of the diseases is not well understood. A whole spectrum of age-dependent pathological features of TDP-43 developed in the motor neurons of the ventral horn in the spinal cord of the male N390D/+ mice was also identified by immunofluorescence (IF) staining (Fig. 3c and d). Since the neurons in the dorsal horn were not motor neurons, we did not focus our analysis on the dorsal horn of the spinal cord. First, anti-ChAT of the spinal cord sections showed that there was an age-dependent loss of spinal cord MN in N390D/+ mice. When compared to the +/+ and A315T/+ mice, the loss was nearly 30% at the age of 6 months, and it increased to 62% at 12 months and 77% at 24 months of age (upper scatter dot plot in Fig. 3d). Second, the level ubiquitinated proteins increased in the ChAT(+) MN of spinal cord of 6-month and older, e.g. 24-month old, mice, and most of which were in the cytosol (Fig. 3c, left lower scatter dot plot in Fig. 3d and Additional file 4: Figure S4c). Thirdly, a portion of the TDP-43 was mislocalized to the cytosol in the spinal cord ChAT(+) MN of 6-month old N390D/+ mice without forming large aggregates (diameter > 2 μm) (Fig. 3c). Larger TDP-43(+) aggregates appeared in the cytosol of spinal cord MN of the older (12-month and 24-month of age) N390D/+ mice, and they were colocalized with the ubiquitinated proteins (Fig. 3c and right lower scatter dot plot in Fig. 3d). Use of different primary antibodies for immunostaining of different samples generated different patterns of aggregate formation, thus excluding the possibility of the presence of signals from auto-fluorescence of lipofuscin. The IF data were supported by Western blotting analysis of fractionated spinal cord extracts. As shown, the fraction of the cytosolic TDP-43 of spinal cord was ~ 15% at the age of 6 months and it increased to ~ 40% by 24 months (Fig. 3e). Furthermore, IF staining of the spinal cord sections showed that there was an age-dependent increase of pTDP-43 in the spinal cord (Additional file 4: Figure S4c) and spinal cord MN (Additional file 4: Figure S4d-g) of N390D/+ male mice. Note that there were also strong signals of Ub and pTDP-43 in the dorsal horn of the spinal cord. Finally, pTDP-43 was also colocalized with ubiquitin partially in the spinal cord MN of 12-month old male N390D/+ mice and this colocalization increased greatly at the age of 24 months (Additional file 4: Figure S4 f and g).

Increasing evidence showed that glial pathology would mediate the disease progression of ALS via reactive astrocytes and microglial activation [52]. In particular, studies of the reactive astrocytes surrounding the degenerating motor neurons in the spinal cord of ALS suggested a crucial role of astrogliosis in ALS pathology [52]. Unlike most of ALS transgenic mouse models displaying strong signal of GFAP in the mouse spinal cord or cortex sections at early stage of pathogenesis refs in review [64], astrogliosis and microgliosis were not seen in the spinal cord sections of 6-month old N390D/+ mice. However, significant astrogliosis was detected in the spinal cord of aged N390D/+ mice (Additional file 5: Figure S5), suggesting the existence of a threshold effect so that the gliosis was moderate in the young N390D/+ male mice.

Where ALS disease begins has remained argumentative. One of the hypotheses is “dying-back”, in which ALS begins within the lower motor neurons (LMN) and then the pathology spreads from LMN to the upper motor neurons (UMN) [15, 17, 66]. Notably, in our study, while there was already 30% loss of motor neurons in the lumbar region of the spinal cord of N390D/+ male mice at the age of 6 months (Fig. 3d), the motor cortex, especially the primary motor cortex, only exhibited significant loss of NeuN(+) neurons at the age of 24 months old but not 3 months and 6 months (Additional file 4: Figure S4 h). This pattern of pathogenesis appeared to be similar to the “dying-back” hypothesis.

#### Alteration of autophagy and proteasome activity

The above data clearly revealed abnormal intracellular localization and aggregation of TDP-43 in the spinal cord of the N390D/+ mice in concert with the mis-regulation of TDP-43 metabolism. The occurrence of TDP-43 protein aggregates was known to be countered by the combined actions of autophagy and the ubiquitin proteasome system (UPS) [57]. We therefore examined the expression patterns of autophagy proteins and the activities of proteasome in the forebrain and the spinal cord. As shown in Fig. 4a, while the ratio of LC3-II/ LC3-I in the spinal cord of 3-month old N390D/+ mice was higher than that of A315T/+ or +/+ mice, it decreased during the post-symptomatic stage to a level significantly lower than those observed in the 6-month old A315T/+ and +/+ mice. In addition, the amount of the classical receptor p62 of autophagy also decreased in the spinal cord, but not the forebrain, of 12-month and 24-month old N390D/+ male mice (Additional file 6: Figure S6, a and b). The proteasome activities in the spinal cord, but not the forebrain, of symptomatic (6- and 24-month old) N390D/+ mice were enhanced when compared to the age-matched +/+ mice and 3-month old N390D/+ mice (Additional file 6: Figure S6, c and d). The data described above together demonstrate the presence of TDP-43 dependent pathology of the autophagy system in the spinal cord of the symptomatic N390D/+ mice.

### Increase of spinal cord Bcl-2 protein as a consequence of mis-regulation of *Bcl-2* pre-mRNA splicing in N390D/+ male mice

Among the regulators of the autophagy pathway is the Bcl-2/Beclin-1 complex [43] and Bcl-2 has been reported it involves in neuron survival [51]. Significantly, while there was no difference between the levels of spinal cord Bcl-2 protein among the newborn of +/+, A315T/+ and N390D/+ male mice, the amount of spinal Bcl-2 of N390D/+ mice was higher than that of the +/+ or A315T/+ mice by approximately 1.5, 2, and 4-fold at the ages of 3, 6, and 12 months, respectively, in parallel to the increase of the spinal TDP-43 protein (Fig. 4b and c, Additional file 7: Figure S7a). *Bcl-2* mRNA is one of the potential neuronal RNA targets of TDP-43 [58], we thus suspected that the higher levels of Bcl-2 protein in the N390D/+ spinal cord might be at least in part due to mis-regulation of *Bcl-2* pre-mRNA splicing. Mouse *Bcl-2* mRNA consists of 2 splicing variants, *Bcl-2*-201 and *Bcl-2-*202 (Ensembl), the former of which encodes the well-studied Bcl-2 protein (NP_033871 from NCBI). Indeed, there was an excellent correlation between the age-dependent increase of spinal Bcl-2 protein and that of the functional *Bcl-2* mRNA, i.e. the isoform 201 in the N390D/+ mice, as shown by RT-PCR analysis (Fig. 4d). The ratio of the *Bcl-2* mRNA isoforms was quantitated using primer 1 and primer 3 in RT-PCR analysis. The data of Fig. 4 c and d strongly suggest that the increase of *Bcl-2* mRNA 201 results from mis-regulation of *Bcl-2* alternative pre-mRNA splicing in the spinal cord of N390D/+ male mice.

To investigate the possibility that the increase of TDP-43 (N390D) plays a causative role in altering the alternative splicing of *Bcl-2* pre-mRNA towards the generation of higher level of *Bcl-2*-201 mRNA, we carried out DNA transfection analysis in mouse Neuro-2a (N2a) cell culture (Fig. 4e). As seen, under the condition of equal amounts of the endogenous TDP-43 vs. exogenously overexpressed mouse wild-type TDP-43, TDP-43 (A315T) and TDP-43 (N390D), respectively, i.e. increase of the total TDP-43 amount in N2a cells by 2 fold, similar to the range of increases (1.5–2.5 fold) of TDP-43 in the spinal cord of 3-month old and 12-month old N390D/+ male mice (Fig. 3b), the endogenous *Bcl-2* mRNA 201 and Bcl-2 protein were elevated only in N2a cells expressing the exogenous TDP-43 (N390D) (Fig. 4e). Taken together, the data from Fig. 4b-e demonstrate that at comparably high levels, only mutant TDP-43 (N390D), but not wild-type or mutant TDP-43 (A315T), increases the expression of *Bcl-2* mRNA isoform 201 as the result of changes in alternative splicing of the *Bcl-2* pre-mRNA.

Besides *Bcl-2*, we also checked the expression patterns of several known TDP-43 target genes. Among them, the splicing pattern of the pre-mRNA of *Eif4h* was altered in mutant TDP-43 transgenic mouse models [1, 3, 65]. We found that the alternative splicing scheme of *Eif4h* pre-mRNA was also mis-regulated in the spinal cord of N390D/+ male mice at the age of 3 months (Additional file 7: Figure S7b) and at the symptomatic stage (data not shown). In addition, similar to changes observed in sporadic ALS patients [55], the mRNA levels of 3 genes, *Ankrd12*, *Mapt*, and *Rbm39,* were decreased in the spinal cord of 3-month old N390D/+ male mice (Additional file 7: Figure S7c) as well as 6-month old ones (data not shown).

### TDP-43 proteinopathies and MN degeneration in culture

**Fig. 5:**
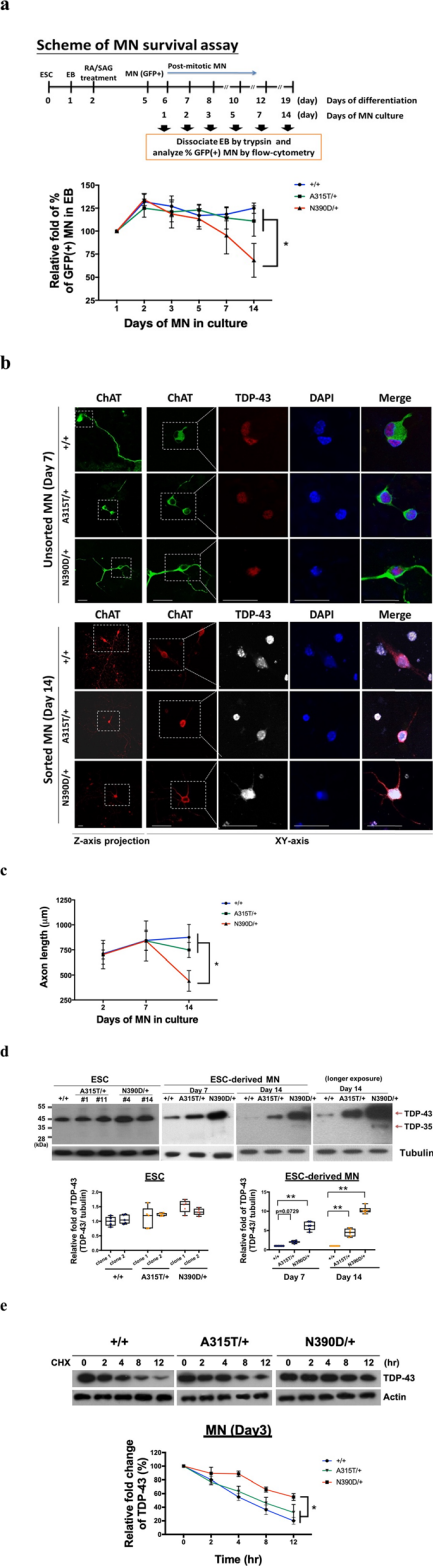
Comparative analysis of patho-signatures and neurodegeneration of cultured spinal cord MN derived from +/+ and mutant ESC. **a** Survival curves of MN in culture. The percentage(s) of GFP (+) cells in the cell mixtures on day 1 of MN culture is defined as 1. The data showed that (N390D/+) MN became more vulnerable to death than (A315T/+) MN or (+/+) MN on day 14 in culture. Mean ± SD *p < 0.05. **b** Immunofluorescence co-staining analysis of MN on day 7 and day 14 in culture using anti-ChAT (green or red) and anti-TDP-43 (red or white). The white boxes in the panels of the first column mark the areas magnified for higher resolution. The axonal morphology under lower magnified field is displayed with Z-axis projection. The scale bars are 50 μm. **c** The statistical analysis of the axon lengths from the WT and mutant MN on different days in culture is shown in the line graph. *N* > 50. *p < 0.05. **d** Comparison of the levels of TDP-43 at early stages of ESC-derived MN in culture (from day 1 to day 3), as analyzed by Western blotting. The statistical analysis is shown in the lower scatter plot (mean ± SD). * *p* < 0.05. **e** The stability of TDP-43 polypeptides in spinal cord (N390D/+) MN, (A315T/+) MN, and (+/+) MN on day 2 in culture by cycloheximide (CHX) assay and Western blotting analysis. The graph below the blots shows that TDP-43 is more stable in (N390D/+) MN in comparison to either (+/+) MN or to (A315T/+) MN. *p < 0.05

To examine whether the differential effects of TDP-43 (N390D and A315T) knock-ins on ALS pathogenesis were due, at least in part, to cell-autonomous effects on spinal cord motor neurons (MN), we generated spinal cord MN in culture. To accomplish this, the N390D/+ and A315T/+ mice were crossed with Hb9:GFP transgenic mice. Embryonic stem cells (ESCs) were derived from TDP-43 (A315T/+)/ Hb9:GFP or TDP-43 (N390D/+)/ Hb9:GFP mice, and then differentiated to GFP(+) MN in culture (Additional file 8: Figure S8). The survival curves as well as the average axonal lengths of the mutant MN were similar to those of (+/+) MN up to 7 days in culture (Fig. 5a-c); however, on day 14 in culture, the survival and average axon length of (N390D/+) MN was significantly reduced in comparison to (+/+) MN or (A315T/+) MN (Fig. 5a-c). Similar to (+/+) MN, the majority of TDP-43 of the mutant MN was confined in the nucleus prior to 7 days in culture, as shown by immunofluorescence staining (upper panels of Fig. 5b). However, the amount of TDP-43 was greater in the cytosol of (N390D/+) MN on day 14 in culture, but without TDP-43 aggregate formation, as shown in the lower panels of Fig. 5b. This observation was similar to that of the symptomatic spinal cord MN of N390D/+ male mice (Fig. 3c). On the other hand, the level of TDP-43 in ESC-derived mutant (A315T/+) MN was 2- to 4-fold higher than the (+/+) MN on day 7 and day 14, respectively. Interestingly, despite of the significant reduction of average axonal lengths of the mutant MN after day 14 in culture, the level of TDP-43 in mutant (N390D/+) MN was 6- and 11-fold higher than (+/+) MN on culture day 7 and day 14, respectively, with the appearance of TDP-35 species on day 14 in culture (Fig. 5d). The higher amount of TDP-43 in mutant MN could be in part due to increased stability of the mutant TDP-43 protein (Fig. 5e). Thus, the analysis of cultured MN derived from ESC suggests that time-dependent, spinal cord MN-autonomous toxic effects underlie the role of the N390D mutation of TDP-43 in age-dependent ALS-like pathogenesis of N390D/+ mice.

#### Increase of *Bcl-2* mRNA and Bcl-2 protein in cultured (N390D/+) MN

**Fig. 6:**
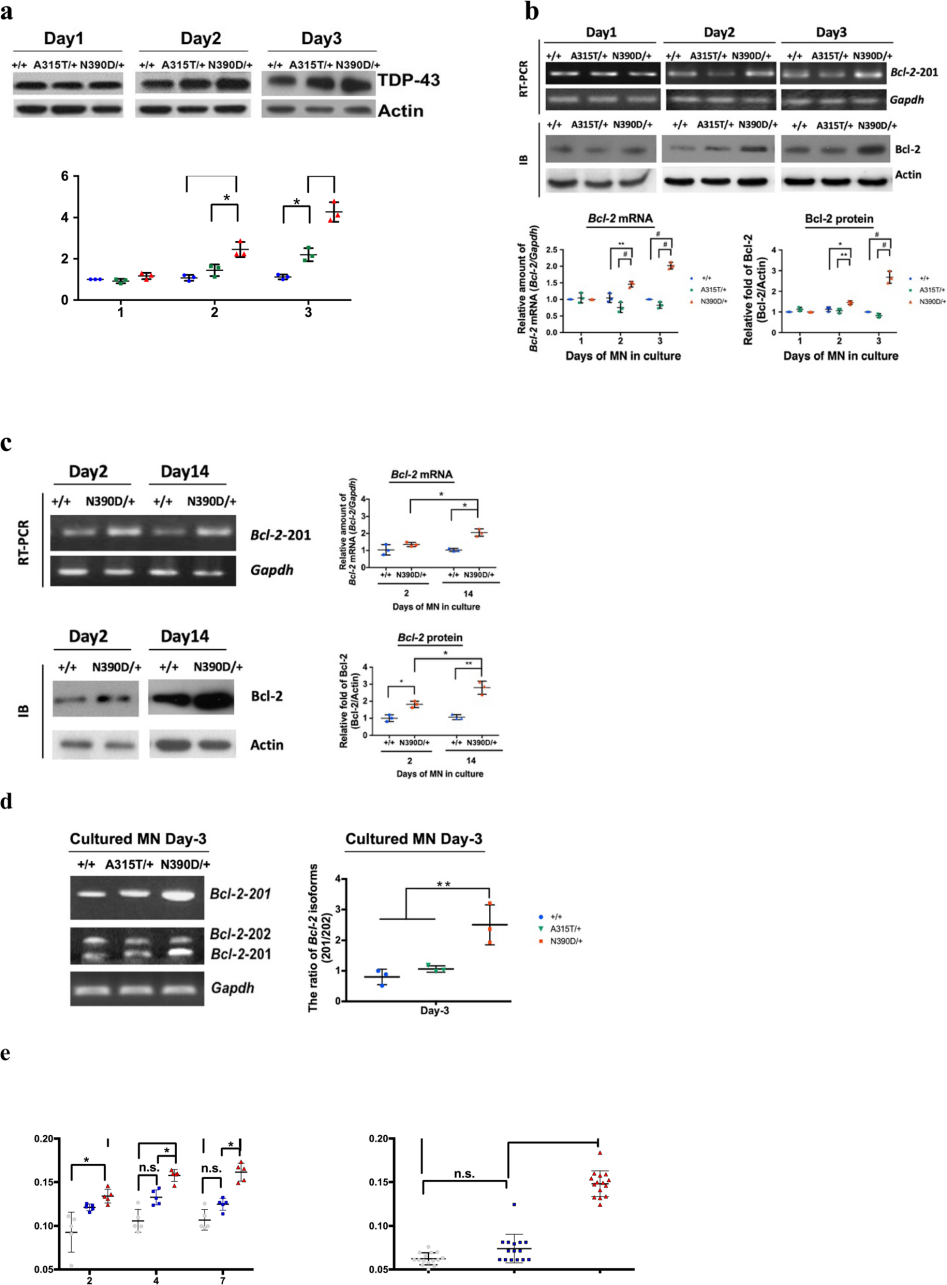
Mis-regulation of Bcl-2 expression and calcium ion homeostasis in cultured (N390D/+) MN. **a** Comparison of the levels of TDP-43 at early stages of ESC-derived MN in culture (from day 1 to day 3), as analyzed by Western blotting. The statistical analysis is shown in the lower scatter plot (mean ± SD). **b** Comparison of the expression levels of *Bcl-2*-201mRNA and Bcl-2 protein as analyzed by RT-PCR (exemplified in the upper panels) and Western blotting (exemplified in the middle panels), respectively. The statistical analysis is shown in the lower scatter plots (mean ± SD). **c** Comparison of the levels of *Bcl-2* mRNA by RT-PCR (upper panel) and Bcl-2 protein by Western blotting (lower panel) and in cultured (+/+) MN and (N390D/+) MN on day 2 and day 14, respectively. **d** RT-PCR detection and statistical analysis (left scatter plot, mean ± SD) of the changes of the relative ratio of the two *Bcl-2* mRNA isoforms. Note the increase of functional *Bcl-2* -201 mRNA in the spinal cord of N390D/+ male mice at both pre-symptomatic (3-month) and symptomatic (6-month) stages. *N* = 3 (randomly chosen from each of the two independent lines) per group. **e** The relative levels of the cytosolic calcium ion levels in cultured MN (left plot) and transfected HEK-293 T cells (right plot) overexpressing human TDP-43(wild-type; WT), TDP-43 (A315T) or TDP-43 (N390D). The relative calcium ion levels were analyzed with the use of Fura2-AM reagent and compared in the separated scatter plots (mean ± SD). Note the overloading of the cytosolic calcium ion in cultured (N390D/+) MN and in HEK-293 T cells overexpressing TDP-43 (N390D) in comparison to TDP-43 (WT) and TDP-43 (A315T). Significantly different represented in **a**-**e**: * *p* < 0.05, ** *p* < 0.01, # *p* < 0.001. n.s., not significant

Significantly, in parallel to the time-dependent increase of the TDP-43 level (Fig. 6a), there was an increase of either *Bcl-2* mRNA 201 or Bcl-2 protein in the cultured (N390D/+) MN (Fig. 6b and c) resulting from mis-regulation of the alternative splicing of *Bcl-2* pre-mRNA (Fig. 6d). On the other hand, the levels of *Bcl-2* mRNA 201 and Bcl-2 protein in (A315T/+) MN were similar compared to those of (+/+) MN on culture after day 2 (Fig. 6b) and day 14 (data not shown), despite of the increase of TDP-43 in cultured (A315T/+) MN (Fig. 6a). The data of Figs. 4 and 6 together indicate that there are cell-autonomous mis-regulation of *Bcl-2* pre-mRNA splicing and consequent increase of Bcl-2 protein in (N390D/+) MN as caused by the increased level of TDP-43. Furthermore, there is a lag between the time on-set of Bcl-2 protein increase and MN degeneration.

#### Changes of calcium ion homeostasis in cultured (N390D/+) MN

Since Bcl-2 is known to increase ER calcium ion leakage resulting in overloading of the cytosolic calcium ion (Ca^2+^) [7, 46], we examined the intracellular level of calcium ion in ESC and ESC-derived spinal MN. As seen, the cytosolic calcium ion concentrations in the (N390D/+) ESC-derived MN was higher than those derived from (+/+) ESC or (A315T/+) ESC (Fig. 6e, left panel), despite similar concentration of calcium ion in (+/+) ESC, (A315T/+) ESC and (N390D/+) ESC (data not shown). Furthermore, overexpression of human TDP-43 (N390D) provoked the intracellular level of calcium ion in HEK293T cells, but not in those cells overexpressing similar amount of either wild-type TDP-43 or TDP-43 (A315T) (Fig. 6e, right panel). A previous study [43] also provided relevant evidence that Bcl-2 was involved in neurotoxicity. In particular, knockdown of Bcl-2 by siRNA in stable clones of HEK-293 cells expressing mutant TDP-43 (A382T) or TDP-43 (M337 V) would decrease the mislocalizaiton of TDP-43 and reduce Ca^2+^ release from ER. Thus, neurotoxicity indeed could be caused by up-regulation of Bcl-2 protein. The above data taken together show that elevation of the cellular level of TDP-43 (N390D) would increase the intracellular cytosolic calcium ion concentration as a result of the increase of Bcl-2 protein.

## Conclusion

The availability of appropriate ALS mouse models is essential for understanding ALS disease mechanisms and the development of therapeutic drugs. Most of previously developed ALS-TDP mouse models are based on a transgenic approach, whereby wild type or mutant TDP-43 is expressed under the control of different promoters [18, 23, 40, 63]. This approach is limited by the cell type specificity of the promoters used to express the wild type and mutant TDP-43 transgenes, so the timing and level of the transgene expression is difficult to control. Furthermore, transgene over-expression causes neurotoxicity and other side effects due to the differences in the timing and level of the transgene expression, even with the wild type TDP-43 gene. Here we describe the use of knock-in strategy to study ALS pathogenesis as a consequence of different ALS-associated TDP-43 mutations. A comparison of the pathologies of two heterozygous mouse models, N390D/+ and A315T/+ that we have generated to those knock-in mouse models reported in literature [24, 26, 72] demonstrate the distinctive pathological effects of different TDP-43 mutants. More importantly, the TDP-43(N390D/+) mice appear to be a genuine ALS-TDP model for further basic and translational research of ALS-TDP.

It is somewhat unexpected but not totally surprising that in the genetic background of C57BL/6 J mice, only N390D but not A315T mutation of TDP-43 exhibits a dominant causative role in ALS-TDP pathogenesis (Fig. 1). A315T is an extensively studied fALS mutation. Analysis of transgenic rodent models or transfected cell cultures have suggested that overexpression of human TDP-43^A315T^ causes neuron degeneration [71] and dosage-dependent cytotoxicity [75], induces ER stress [67], and affects neuronal mitochondrial morphology [70]. However, mice expressing human TDP-43^A315T^ under the control of endogenous mouse *Tarabp* promoter develop mitochondria dysfunction but without obvious others phenotypes [61]. It has been suggested that variations in the ALS-TDP pathological phenotypes among the ALS-TDP patients result from their different genetic background as well as from environmental factors [9, 77]. The lack of ALS pathology of this mouse model and our A315T mice could reflect the differential effects of the mouse and human genetic backgrounds on the development of ALS pathogenesis. It is also worthy to note that the N390D/+ mice and A315T mice investigated in the current study are all in the B6 background. Their phenotypes are likely to change under a different genetic background, as shown before for several other genetically modified neurodegenerative disease mouse models, e.g. the transgenic AD mouse model 5xFAD (database, by Jackson Lab) and the mice carrying TDP-43 mutations induced by ENU [26].

**Fig. 7:**
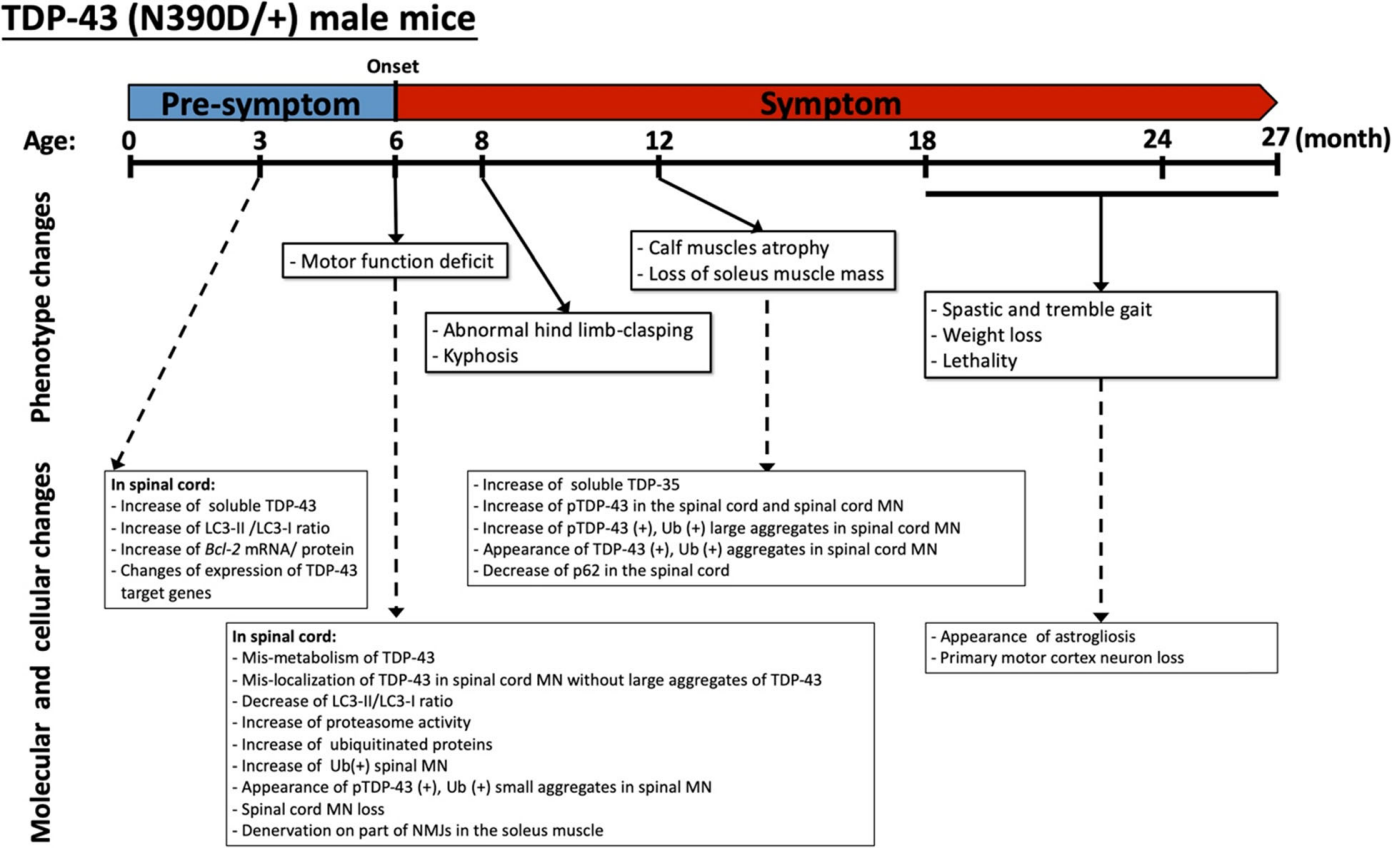
Age-dependent ALS-TDP-like pathogenesis of TDP-43 (N390D/+) male mice. The timeline (month) illustrates the major morphological, behavioral, cellular, and molecular events during the pre-symptomatic and symptomatic stages of heterozygous male TDP-43 (N390D/+) mice. For more details, see text

In striking contrast to heterozygous A315T (this study), Q331K [72], M337 V and G298S [24] knock-in mice, the heterozygous N390D/ + male mice develop molecular, cellular, and behavioral changes, with a spectrum of ALS-like phenotypes that appear at the age the 6 months and then progress (Fig. 7). These phenotypes include shortened life span (Fig. 1b), body weight loss of the remains (Fig. 1c), age-dependent motor dysfunction (Fig. 2a), kyphosis (Fig. 2c), muscle atrophy (Fig. 2d and e) and denervation of NMJ (Fig. 2f). The lack of body weight loss in N390D/+ mice is similar to the Q331K knock-in mouse model the basis of which has been attributed to hyperphagia and transcriptomic changes [72]. Notably, other TDP-43 disease models have been shown to have increased body fat, decreased lean muscle mass, and larger adipocytes in white fat [60]. Therefore, we guess that maintenance of the body weight in N390D/+ mice might also be due to some of these effects. In the case of the N390D/+ male mice, good care of the diseased mice (see Methods) likely also has contributed to the maintenance of their body weight throughout life.

Accompanied with these phenotypes are various molecular and cellular pathologies (Fig. 7) including the progressive increases of spinal cord TDP-43/ TDP-35/ TDP-25 (Fig. 3a and b), insoluble TDP-43 species (Fig. 3b), pTDP-43 (Additional file 4: Figure S4), and the accumulation of cytosolic TDP-43/ pTDP-43 aggregates in the spinal cord MN of symptomatic N390D/+ male mice (Fig. 3c and d, and Additional file 4: Figure S4, d-g). Ubiquitinated proteins also increased in the spinal cord MN and they are partially colocalized with the cytosolic TDP-43/ pTDP-43 aggregates (Fig. 3c and d, and Additional file 4: Figure S4, d-g). The reactive astrocytes surrounding the degenerating motor neurons in the spinal cord of ALS have suggested a crucial role of astrogliosis in ALS pathology [52]. However, astrogliosis could only be detected in the spinal cord of N390D/+ mice at late stage of pathogenesis (Additional file 5: Figure S5). It is noteworthy that similar to the heterozygous Q331K/+ knock-in mice [26, 72], N390D/+ male mice still have an average age of ~ 19 months despite of the loss of 30% of their spinal MN already at the age of 6 months and neurodegeneration of the primary motor cortex at the ages of 12 to 24 months. Besides the possible effects of the B6 genetic background, good care of the mice (see Methods) should also have contributed to the life span of N390D/+ male mice.

There are also progressive changes of autophagy and UPS in the spinal cord of N390D/+ mice. Interestingly, the LC3-II/LC3-I ratio in pre-symptomatic (3-month old) N390D/+ male mice is higher than that of the wild-type male mice (Fig. 4a), which we speculate to be due to an initial autophagy response to the TDP-43 mis-metabolism [54]. In any case, the decreased autophagy function in the spinal cord of symptomatic N390D/+ mice, as reflected by the decreased ratio of LC3-II/ LC3-I (Fig. 4a) and p62 level (Additional file 6: Figure S6), would contribute to the pathology progression and eventual death of the spinal cord MN (Fig. 3c and d). On the other hand, there is an enhancement of UPS in the spinal cord during pathogenesis of the N390D/+ male mice, as reflected by the proteasome activity assay (Additional file 6: Figure S6c and d). This increase of UPS in the symptomatic spinal cord of N390D/+ male mice could compensate for the down regulation of autophagy as suggested before for other systems [68].

There is an autoregulation program for the control of the amount of the TDP-43 protein through regulation of the *Tardbp* mRNA decay and/or processing [5, 35]. However, the TDP-43 protein accumulates in the spinal cord MN of N390D/+ male mice in part due to increased stability of the TDP-43 (N390D) polypeptide (Fig. 5e) and Wu et al., 2013 [75] but without obvious change of the *Tardbp* mRNA level (Additional file 3: Figure S3, a and b). This is in contrast to the increase of the *Tardbp* mRNA in the Q331K knock-in mouse model [72], the RRM2mut plus LCDmut ENU mouse models [26] as well as the TDP-43 (A315T) transgenic mouse model [4]. Noteworthy, normal autoregulatory function was also observed in knock-in mouse models carrying ALS-associated M337 V or G298S mutation [24]. In addition, human iPSCs carrying different TDP-43 mutations exhibited a wide range of their *Tardbp* mRNA levels [25]. Thus, different ALS-associated mutations in the C-terminal glycine-rich domain exert differential effects on the autoregulation of TDP-43 protein via metabolism of the *Tardbp* mRNA.

Of particular interest and importance is the finding of the concomitant increase of the levels of TDP-43 and the *Bcl-2* mRNA 201 encoding the 26 kDa Bcl-2 protein in the spinal cord of 3-month, 6-month and 12-month old, but not the newborn, N390D/+ male mice (Fig. 4b and c) as well as in cultured spinal cord (N390D/+) MN at differentiation day 2 and beyond (Fig. 6a-c). Bcl-2 is an anti-apoptotic protein that promotes the survival of neurons [51] and other types of cells [31]. It is also known to affect autophagy [43], intracellular calcium ion homeostasis [7], and consequently the associated cell-fate determining pathways [22, 29] in a dose-dependent manner. Whether a similar effect of TDP-43(N390D) on *Bcl-2* RNA processing operated in human awaits to be seen in future. Also, the causative effect by elevated TDP-43 (N390D) protein on the alteration of *Bcl-2* pre-mRNA splicing and consequent increase of the Bcl-2 protein was not observed for TDP-43(A315T) (Fig. 4c-e), and TDP-43 (A382T) or TDP-43 (M337 V) [54]. Whether it could also be exerted by other ALS-associated TDP-43 mutations is unknown at the present time.

Overall, our data indicate that spinal cord-specific increase of TDP-43 (N390D), due in part to its higher stability, and the associated of gain-of-toxicity in *Bcl-2* pre-mRNA splicing are among the essential early causative events of ALS pathogenesis of the TDP-43 (N390D/+) mice. This leads to upregulation of the Bcl-2 protein, change of autophagy, mis-metabolism of TDP-43 and other proteins, ER stress, etc., all of which would contribute to the age-dependent cytotoxicity/ death of the spinal cord MN and other age-dependent ALS-like phenotypes TDP-43 (N390D/+) mice.

The establishment and comparative analysis of the two mouse models, TDP-43 (N390D/+) and TDP-43 (A315T/+), suggest that different human ALS-associated TDP-43 mutations display distinct pathophysiological changes in mice. The TDP-43 (N390D/+) mice should provide an excellent model to study in detail the initiation and propagation of ALS-TDP under normal physiological conditions. It would also be ideal for use in the validation of potential therapeutic approaches and reagents for the treatment of ALS-TDP.

## Supplementary information

**Additional file 1: Figure S1.** Effects of N390D mutations on the survival of newborn pups and on motor function/ lifespan of N390D/+ female mice.

**Additional file 2: Figure S2.** Cognition analysis of N390D/+ male mice.

**Additional file 3: Figure S3.** Similar levels of *Tardbp* mRNA expression in different tissues of +/+, A315T/+ and N390D/+ male mice.

**Additional file 4: Figure S4.** Increase of phosphorylated TDP-43 (pTDP-43) in the spinal cord, and loss of NeuN(+) neurons of primary motor cortex of the old N390D/+ male mice.

**Additional file 5: Figure S5.** Increase of astrogliosis in the spinal cord of N390D/+ male mice at late stage of pathogenesis.

**Additional file 6: Figure S6.** Decreased of p62 protein level and enhanced of the proteasome activities in the spinal cord, but not the forebrain, of symptomatic N390D/+ mice.

**Additional file 7: Figure S7.** Expression change of *Bcl-2* and other TDP-43 target genes in the spinal cord of N390D/+ male mice in comparison to the +/+male mice.

**Additional file 8: Figure S8.** Scheme of MN differentiation.

**Additional file 9: Movie S1.** 18-month old N390D/+ mouse (#108). Note the 18-month old N390D/+ mice (Additional file 9: Movie S1 and Additional file 10: Movie S2) but not +/+ littermate (Additional file 11: Movie S3) exhibit spastic and tremble gait. Some of them show difficult movement due to paralysis of the hind limbs (Additional file 10: Movie S2).

**Additional file 10: Movie S2.** 18-month old N390D/+ mouse (#361). Note the 18-month old N390D/+ mice (Additional file 9: Movie S1 and Additional file 10: Movie S2) but not +/+ littermate (Additional file 11: Movie S3) exhibit spastic and tremble gait. Some of them show difficult movement due to paralysis of the hind limbs (Additional file 10: Movie S2).

**Additional file 11: Movie S3.** 18-month old +/+ littermate of N390D/+ mouse (#361). Note the 18-month old N390D/+ mice (Additional file 9: Movie S1 and Additional file 10: Movie S2) but not +/+ littermate (Additional file 11: Movie S3) exhibit spastic and tremble gait. Some of them show difficult movement due to paralysis of the hind limbs (Additional file 10: Movie S2).
