# Supplementary material for: A robust TDP-43 knock-in mouse model of ALS
Source: Acta Neuropathol Commun. 2020 Jan 21;8:3. doi: 10.1186/s40478-020-0881-5 (PMC6975031; doi:10.1186/s40478-020-0881-5)
Supplement: Supplementary file 1 — Additional file 1: Figure S1. Effects of N390D mutations on the survival of newborn pups and on motor function/ lifespan of N390D/+ female mice. [file 40478_2020_881_MOESM1_ESM.docx]

**Additional file figures**

**a b**

**
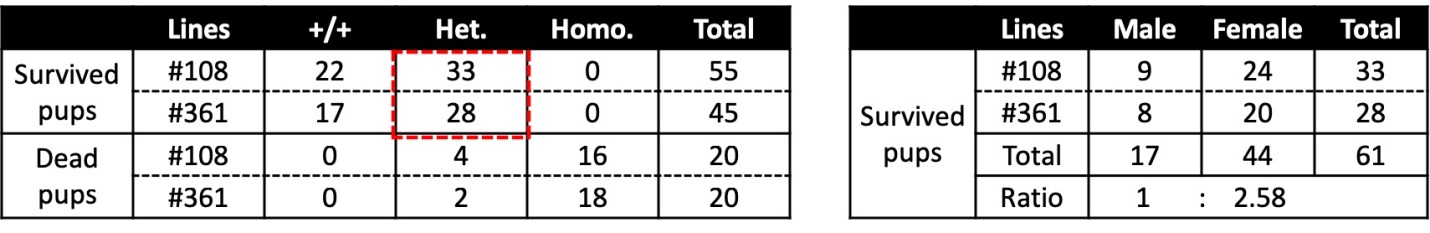
**

**c**

**
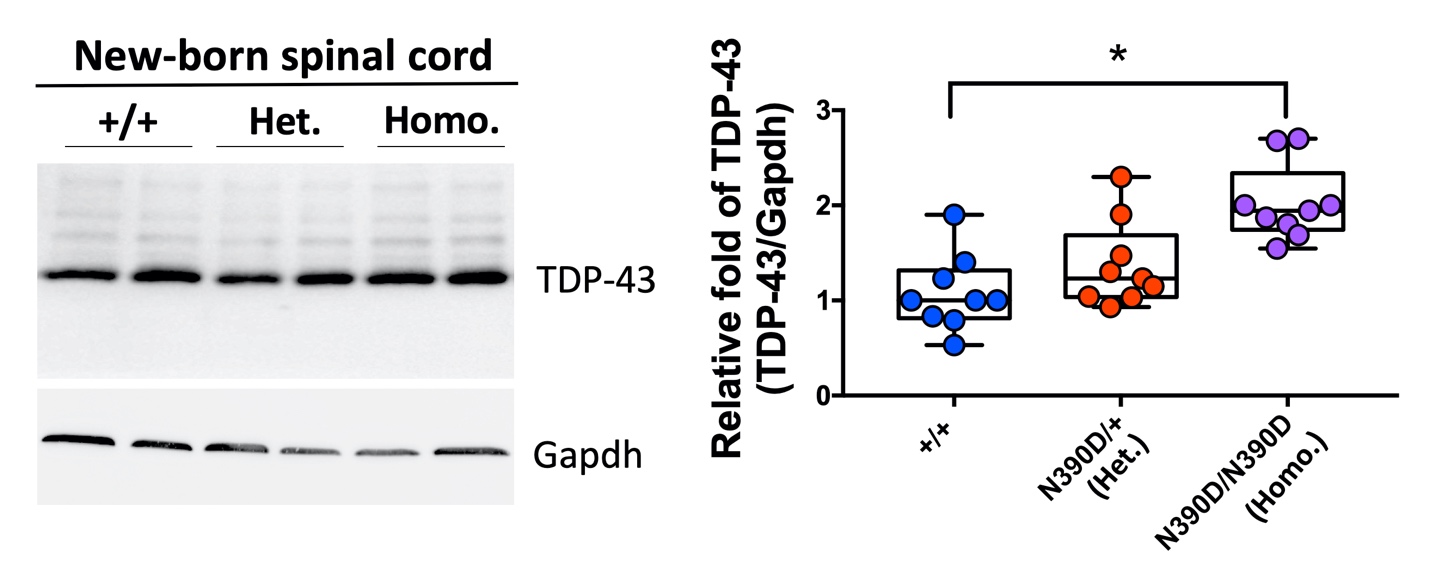
**

**
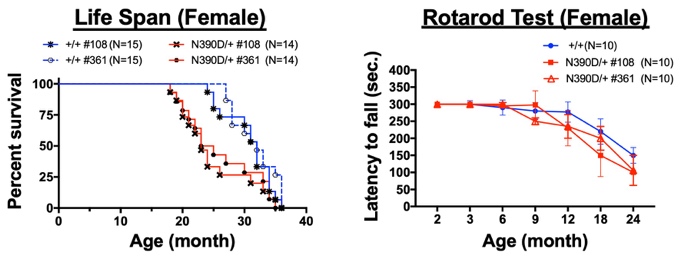
**

**d**

**
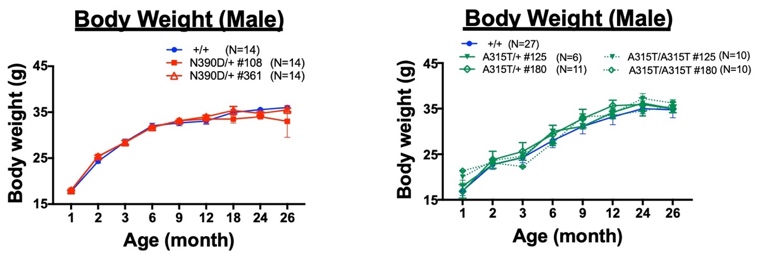
**

**e**

**Figure S1. Effects of N390D mutations on the survival of newborn pups and on motor function/ lifespan of N390D/+ female mice.**

**(a)** Comparison of the numbers of the survived and dead pups of different genotypes from intercrosses #108× #108 and #361× #316, respectively. Note the lethality effect of the N390D mutation on the homozygous N390D/N390D pups (Homo.) but not frequently on the heterozygous N390D/+ pups (Het.). **(b)** Gender distribution of the survived offspring from the red dashed box in (a). **(c)** Comparison of the levels of TDP-43 in the new-born spinal cord of +/+, N390D/+ (Het.) and N390D/N390D (Homo.) mice, as analyzed by Western blotting. The blots are exemplified on the left and the statistical analysis is shown on the right. Note the significant increase of TDP-43 in the spinal cord of male N390D/N390D mice in comparison to +/+ littermates. **(d)** Survival curves (left panel) and rotarod test (right panel) of heterozygous N390D/+ knock-in female mice in comparison to the +/+ female mice. Note the relatively high variations of motor capabilities of the individual female N390D/+ mice in comparison to the +/+, and around 30% of the N390D/+ female mice have similar life span curves as their +/+ littermates. Mean ± SEM. The numbers of analyzed mice (N) are listed in the figure. **(e)** The body weight of N390D/+ male mice (left panel) as well as A315T/+ plus A315T/A315T male mice (right panel) were measured at different ages and compared to the +/+ male mice. Mean ± SEM. The number of mice analyzed per group are listed in the figure.
