# Supplementary material for: A robust TDP-43 knock-in mouse model of ALS
Source: Acta Neuropathol Commun. 2020 Jan 21;8:3. doi: 10.1186/s40478-020-0881-5 (PMC6975031; doi:10.1186/s40478-020-0881-5)
Supplement: Supplementary file 2 — Additional file 2: Figure S2. Cognition analysis of N390D/+ male mice. [file 40478_2020_881_MOESM2_ESM.docx]

**a**

**
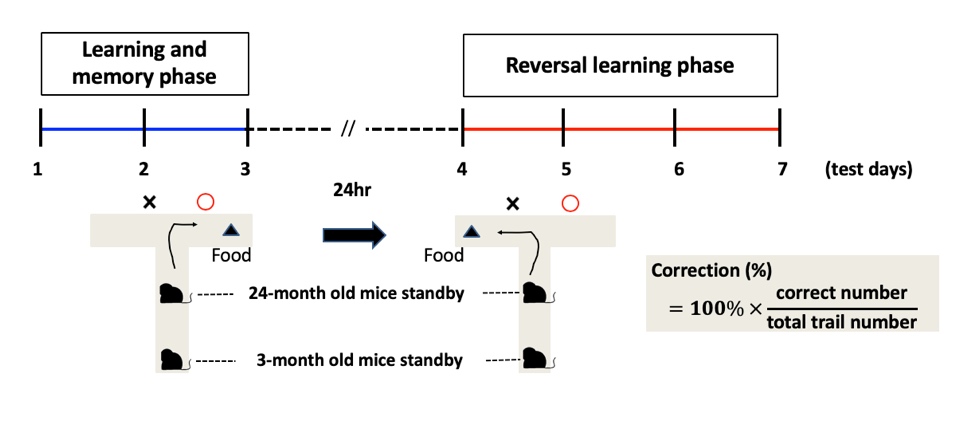
**

**
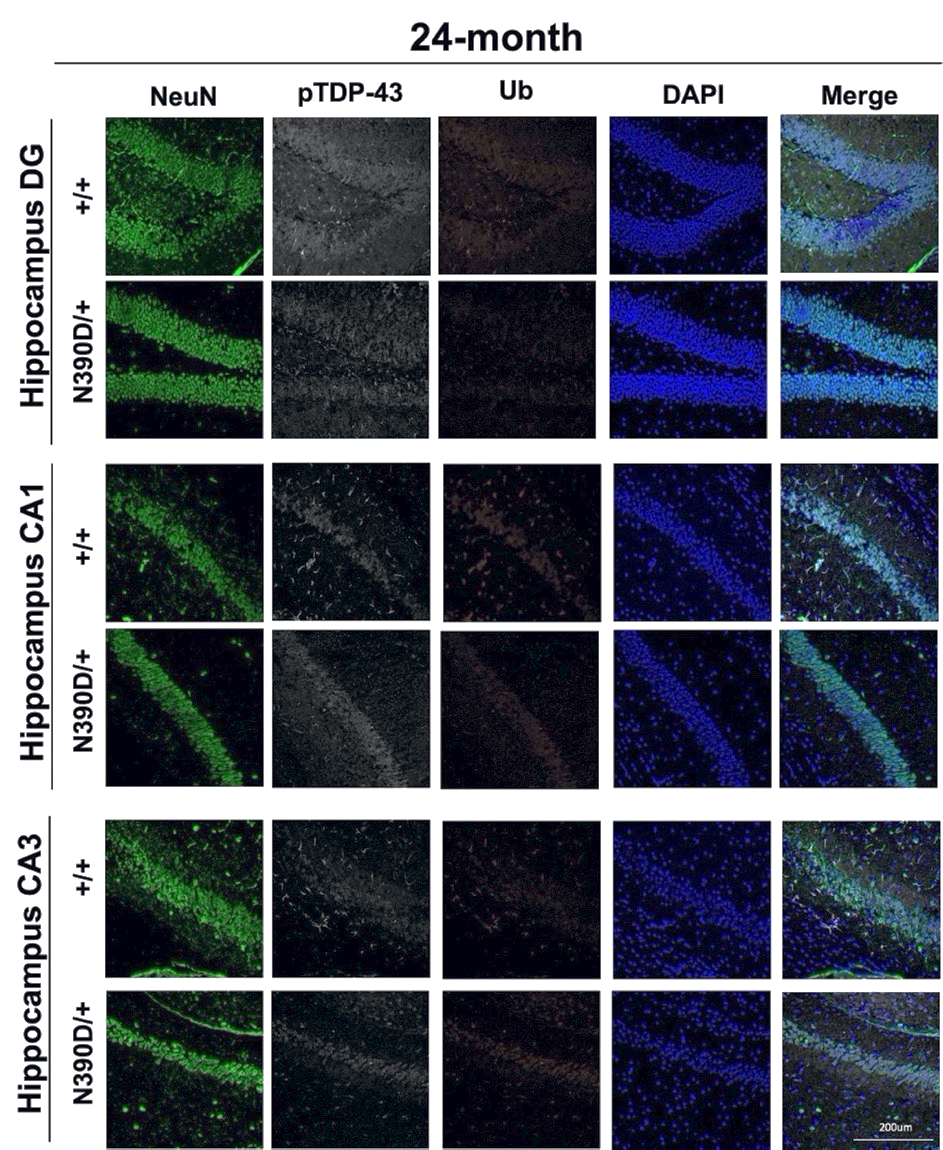

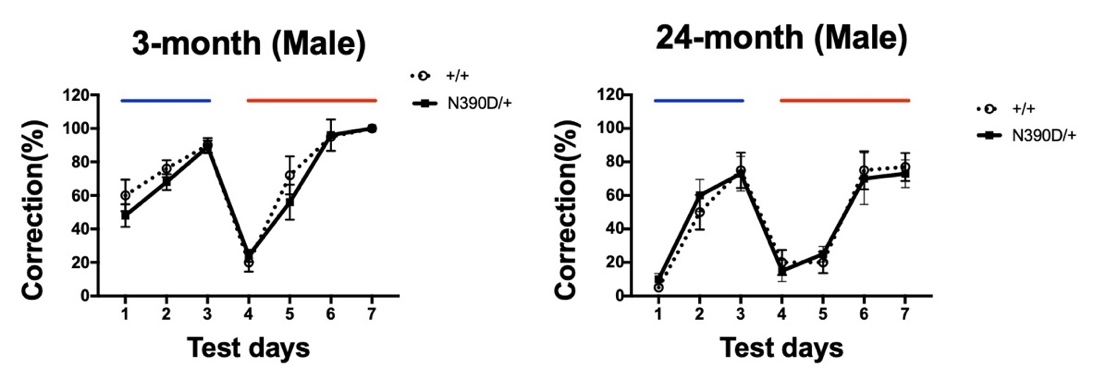
**

**b**

**c**

**
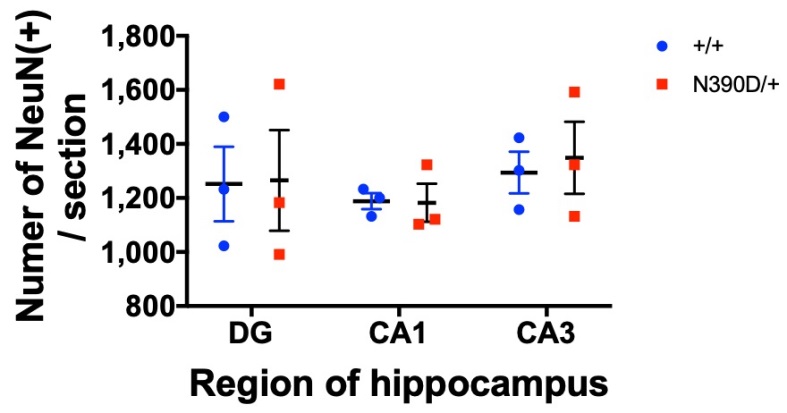
**

**Figure S2. Cognition analysis of N390D/+ male mice.**

**(a)** Flow chart of T-maze test. The mice learned and memorized the position of food during day1 to day3 (blue line). The position of food was changed on day 4 to observe the reversal learning ability of the mice (red line). The marks O and X were used for strengthening the spatial memory of hippocampus. The standby site of the young mice was 30-35 cm from the intersection. The site of 24-month old mice was closer (10 cm) from the intersection because of their dyskinesia. For more details, see Methods. T-maze test results are presented in the lower two panels. Note the similar cognitive function of the N390D/+ male mice as the +/+ male mice at both the ages of 3 months and 24 months (mean ± SEM). N=9 (>3 each from lines #108 and #361) per group. (**b)** Immunofluorescence co-staining analysis of the hippocampus of 24-month old mice using anti-NeuN (green, a neuron marker), anti- pTDP-43 (gray) and anti-ubiquitin (Ub, red). DAPI (blue) indicates the locations of the nuclei. The scale bar is 200 μm. (**c)** Statistical comparison of the average numbers of the neurons per brain section in the hippocampal dentate gyrus (DG), CA1, and CA3 regions of N390D/+ and +/+ male mice (mean ± SEM), respectively. At least 2 discontinuous forebrain sections were counted for each mouse. N=3 (randomly chosen from lines #108 and/or #361) per group.
