# Supplementary material for: A robust TDP-43 knock-in mouse model of ALS
Source: Acta Neuropathol Commun. 2020 Jan 21;8:3. doi: 10.1186/s40478-020-0881-5 (PMC6975031; doi:10.1186/s40478-020-0881-5)
Supplement: Supplementary file 3 — Additional file 3: Figure S3. Similar levels of Tardbp mRNA expression in different tissues of +/+, A315T/+ and N390D/+ male mice. [file 40478_2020_881_MOESM3_ESM.docx]

**a**


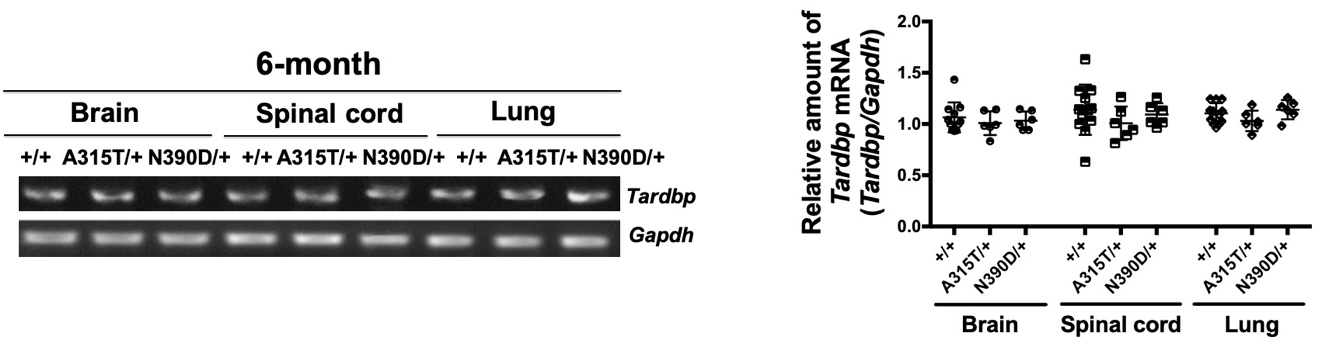


**b**


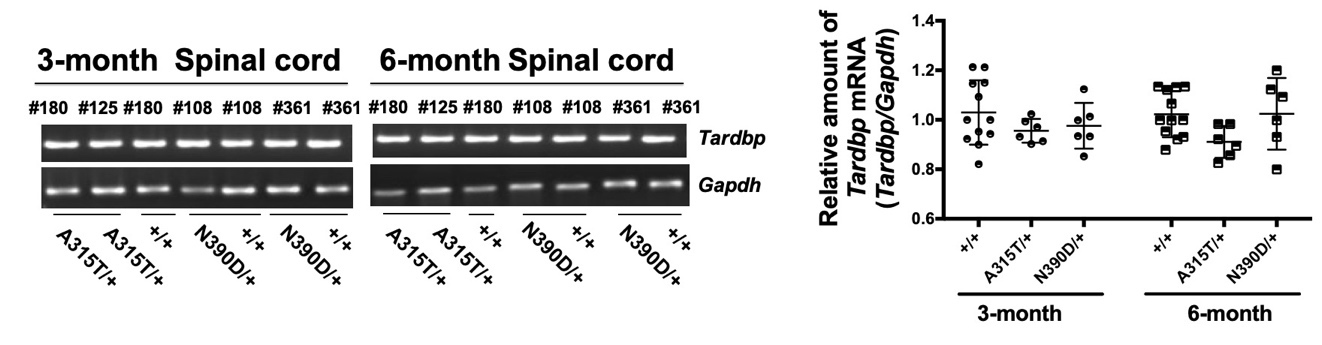


**c**


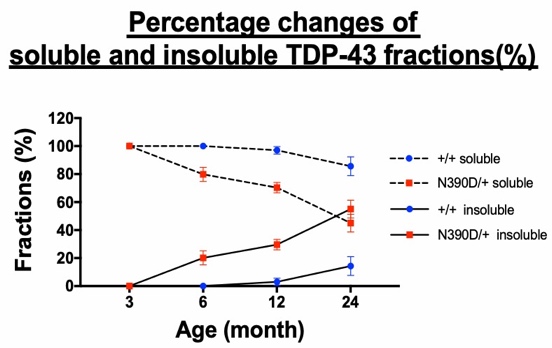


**Figure S3. Similar levels of *Tardbp* mRNA expression in different tissues of +/+, A315T/+ and N390D/+ male mice.**

**(a)** The RT-PCR patterns of *Tardbp* mRNAs in the brain, spinal cord and lung of 6-month old mice. **(b)** The RT-PCR patterns of *Tardbp* mRNAs in the spinal cord from 3-month and 6-month old mice. The corresponding comparisons of the relative levels of *Tardbp* mRNAs by qRT-PCR are shown in the two dot plots on the right side (mean± SD). The number of mice of each group and their line origins are as follows: N=12 for +/+ (3 from each of the independent lines); N=6 (3 from each of the two independent lines) per genotype group. **(c)** The connecting-line plot deduced from Figure2b presents the changes of the fractions (%) of the soluble and insoluble TDP-43 in the spinal cord of N390D/+ and +/+ male mice, respectively. Mean± SD and *p<0.05, **p<0.01.
