# Supplementary material for: A robust TDP-43 knock-in mouse model of ALS
Source: Acta Neuropathol Commun. 2020 Jan 21;8:3. doi: 10.1186/s40478-020-0881-5 (PMC6975031; doi:10.1186/s40478-020-0881-5)
Supplement: Supplementary file 4 — Additional file 4: Figure S4. Increase of phosphorylated TDP-43 (pTDP-43) in the spinal cord, and loss of NeuN(+) neurons of primary motor cortex of the old N390D/+ male mice. [file 40478_2020_881_MOESM4_ESM.docx]

**
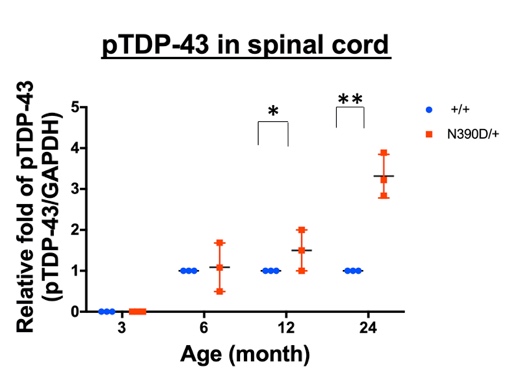
a**

**
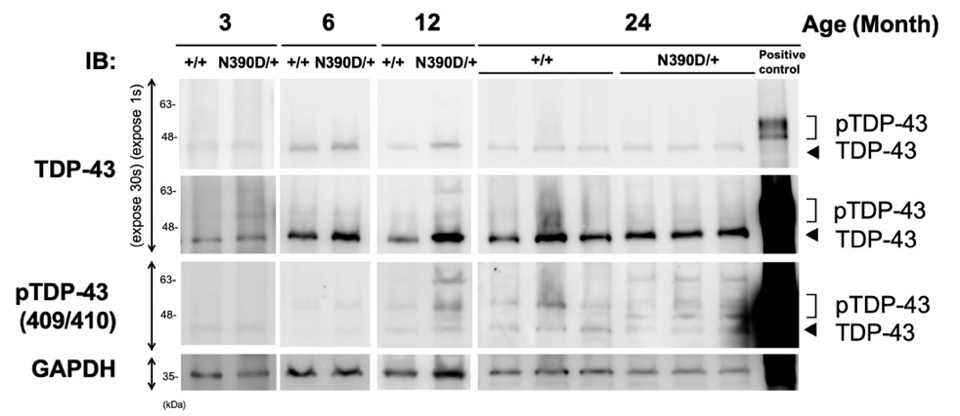
**

**
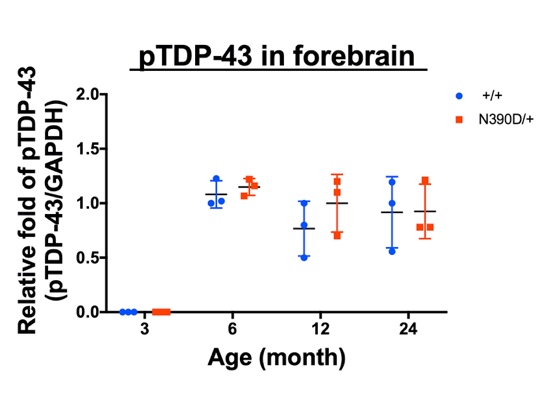
**

**
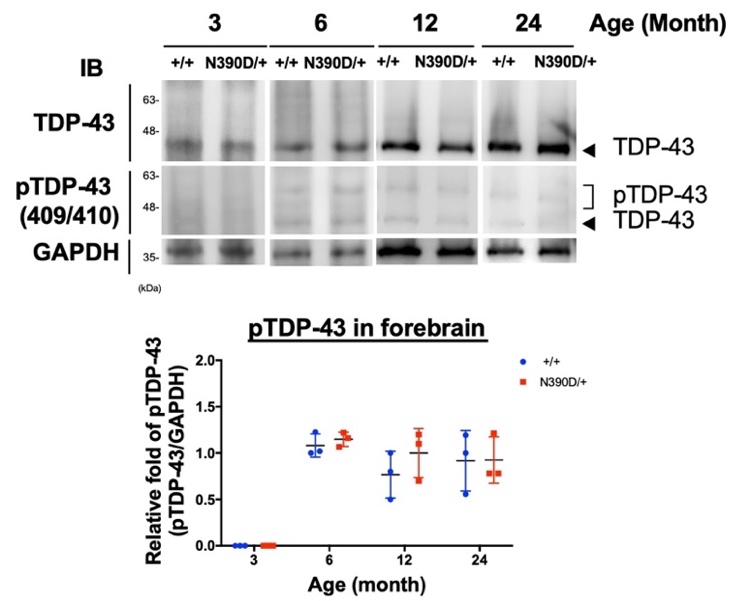
b**

**c**

**
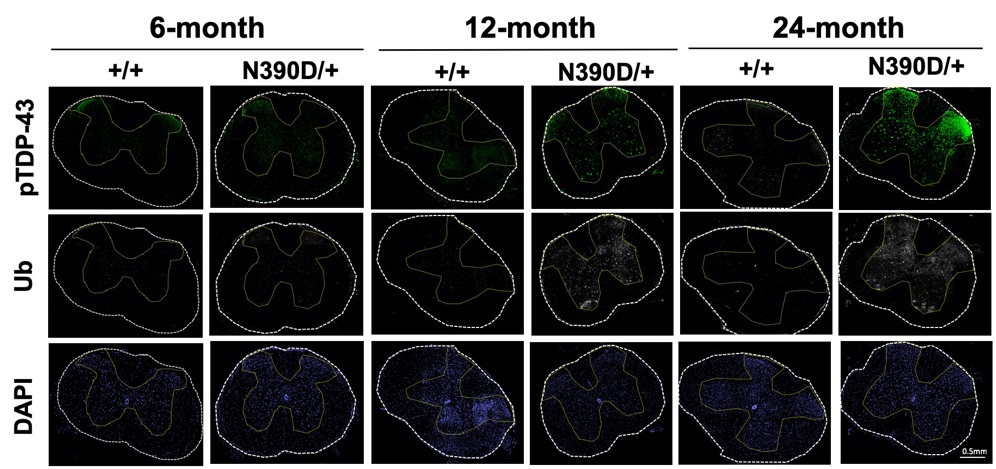
**

**d**

**
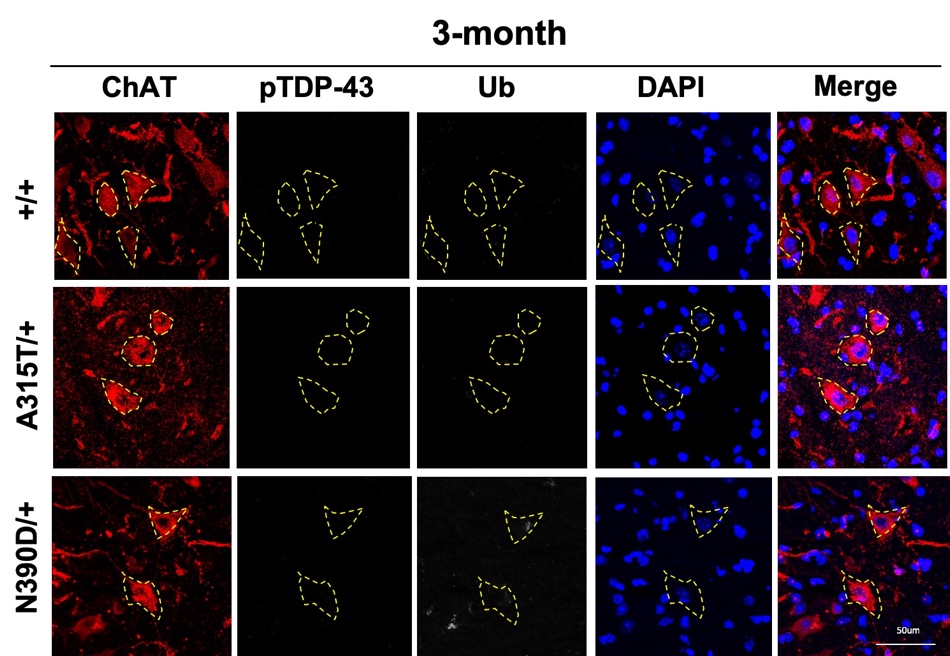
**

**e**

**
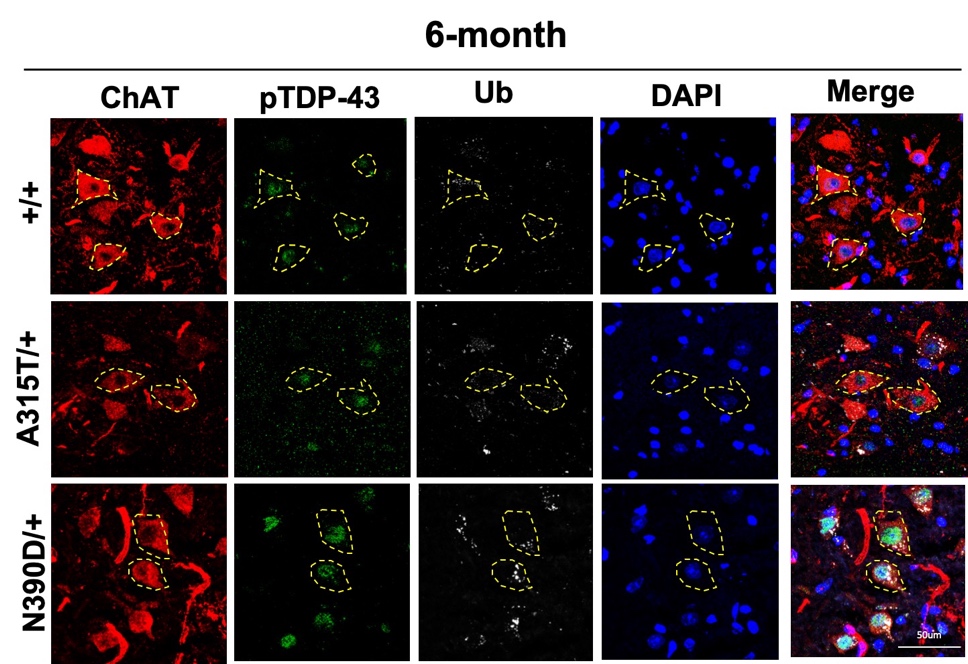
**

**f**

**
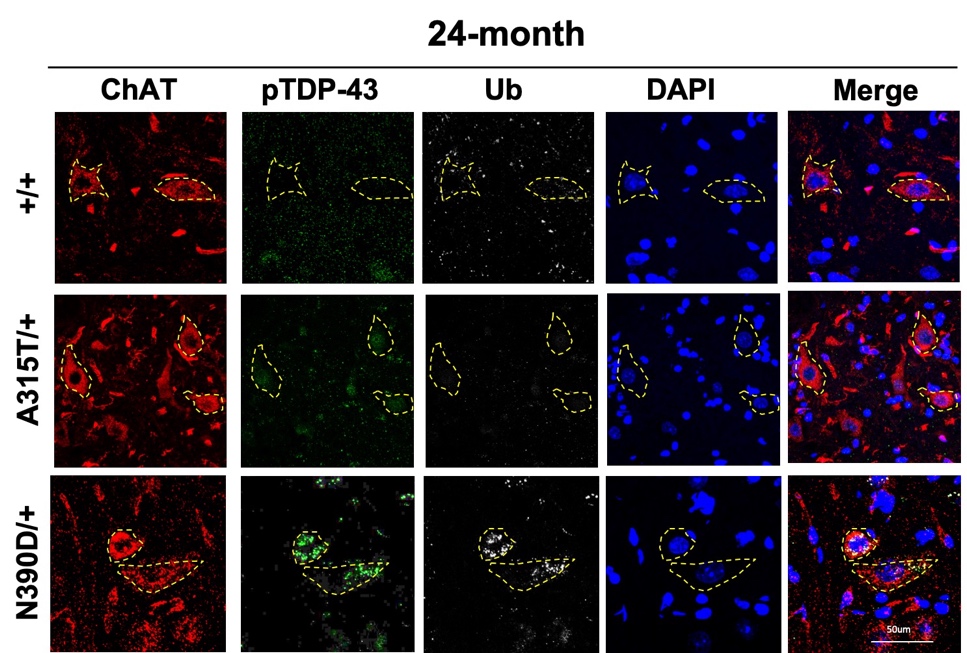
**

**g**

**
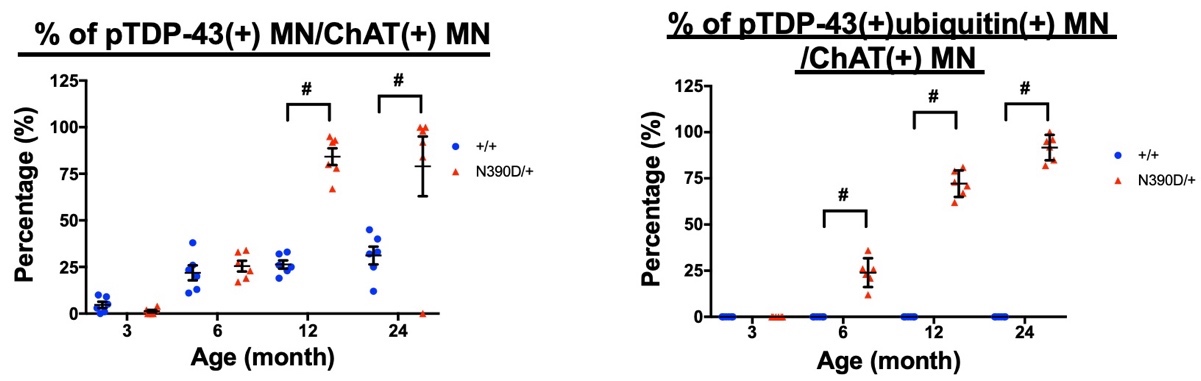
**

**h**

**
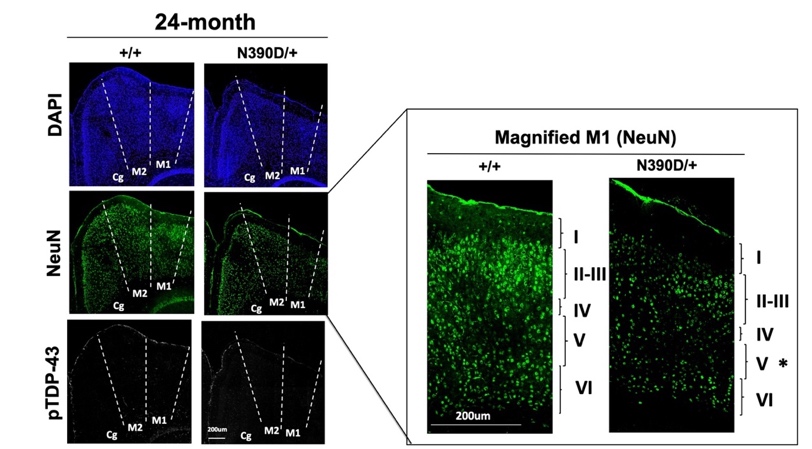
**

**Figure S4. (a-b)** Western blotting analysis of spinal cord extracts **(a)** and the forebrain **(b)** of mice of different ages using anti-TDP-43, anti-pTDP-43(409/410) and anti-GAPDH. The arrowheads indicate the positions of the TDP-43, and the brackets indicate the positions of pTDP-43 on the blot(s). The scatter plot showing the relative levels of the spinal cord pTDP-43 after normalization against GAPDH deduced from the Western blotting data (mean± SD). N=3 (randomly chosen from each of the two independent lines) per group. **p<0.01. **(c)** Immunofluorescence co-staining of pTDP-43 (green) and ubiquitin (Ub; gray) of spinal cord lumbar sections from +/+ and N390D/+ male mice at the age of 6, 12, and 24 months, respectively. The whole view of representative spinal cord sections is presented. DAPI (blue) indicates the locations of the nuclei. Note the significant increase of pTDP-43 and ubiquitin signals in samples from 12- and 24-month old N390D/+ male mice. At least 2 discontinuous sections of lumbar spinal cord were examined for each mouse. N=3 (randomly chosen from each of the two independent lines) per group. **(d-f).** Higher magnification pictures of spinal cord sections co-stained with anti-pTDP-43 (green), anti-ubiquitin (Ub, gray) and anti-ChAT (red). The yellow dashes label the locations of MN. DAPI (blue) indicates the locations of the nuclei. N≧3 (randomly chosen from each of the two independent lines) per group. The scale bars are 50 μm. Note the increase of pTDP-43 signals colocalized with the Ub in the 24-month old N390D/+ mouse samples. **(g)** The comparisons of the % of pTDP-43(+) MN (left) and the % of pTDP-43(+) Ub(+) ChAT(+) MN (right), are represented by the scatter dot plots. At least 2 discontinuous sections of lumbar spinal cord were counted and averaged for each mouse. #p<0.001. **(h)** Immunofluorescence co-staining of mouse brain sections from the motor cortex of +/+ and N390D/+ male mice at the age of 24 months using anti-NeuN (green), anti-pTDP-43 (gray), and DAPI (blue). Cg, cingula cortex; M1, primary motor area; M2, secondary motor area. A part of the image of the M1 area is magnified and shown on the right with the six layers (I-VI) indicated. Note the significant loss of NeuN (+) neurons and decrease of the thickness of the layer 5 (*) in the M1 region of N390D/+ male mice in comparison to the +/+ male mice. At least 2 discontinuous forebrain sections were examined for each mouse. N=3 (randomly chosen from lines #108 and/or #361) per genotype group. Scale bars, 200 μm.
