# Supplementary material for: A robust TDP-43 knock-in mouse model of ALS
Source: Acta Neuropathol Commun. 2020 Jan 21;8:3. doi: 10.1186/s40478-020-0881-5 (PMC6975031; doi:10.1186/s40478-020-0881-5)
Supplement: Supplementary file 5 — Additional file 5: Figure S5. Increase of astrogliosis in the spinal cord of N390D/+ male mice at late stage of pathogenesis. [file 40478_2020_881_MOESM5_ESM.docx]

**a**

**
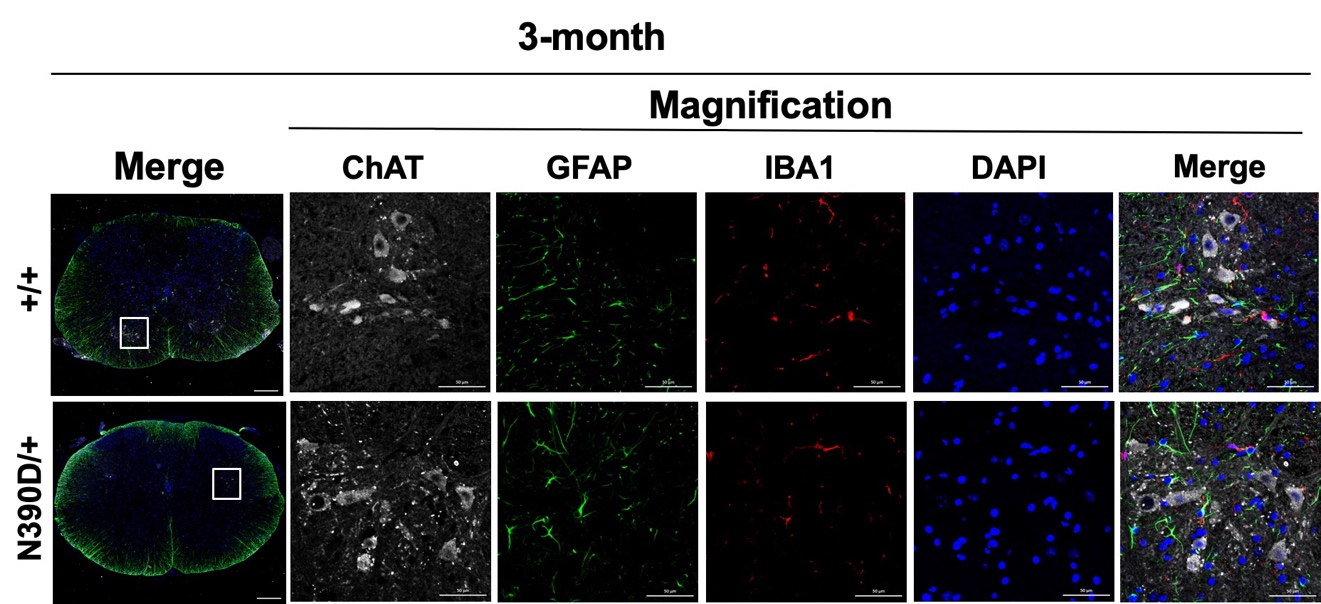
**

**
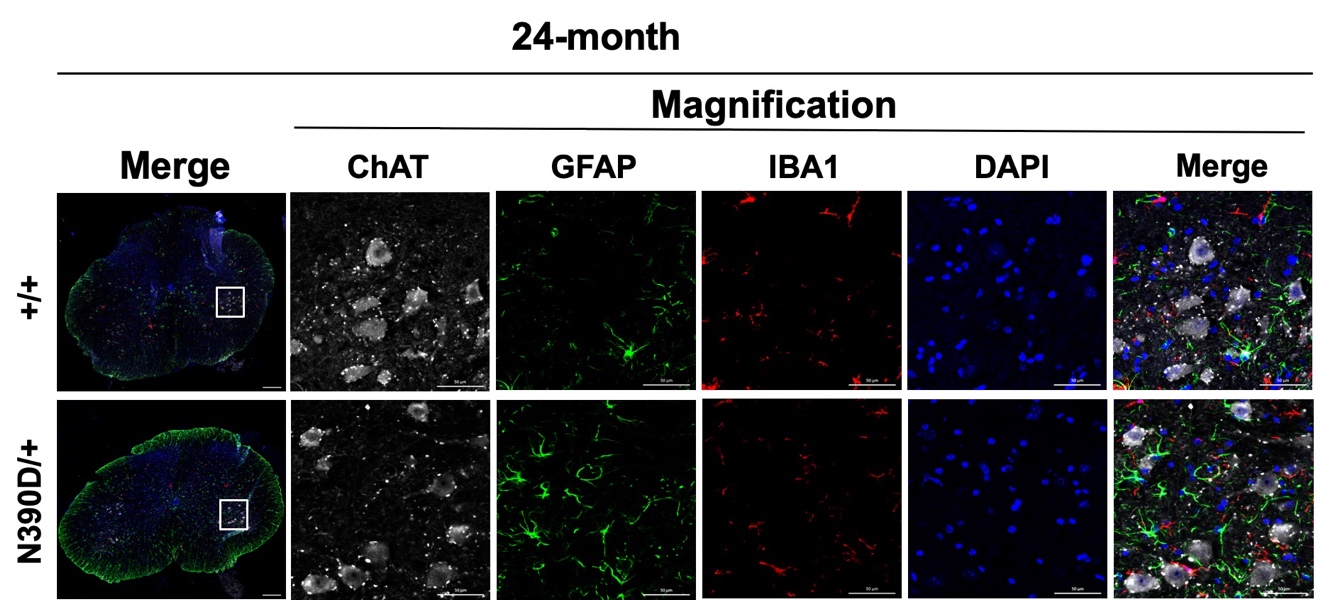
**

**b c**

**
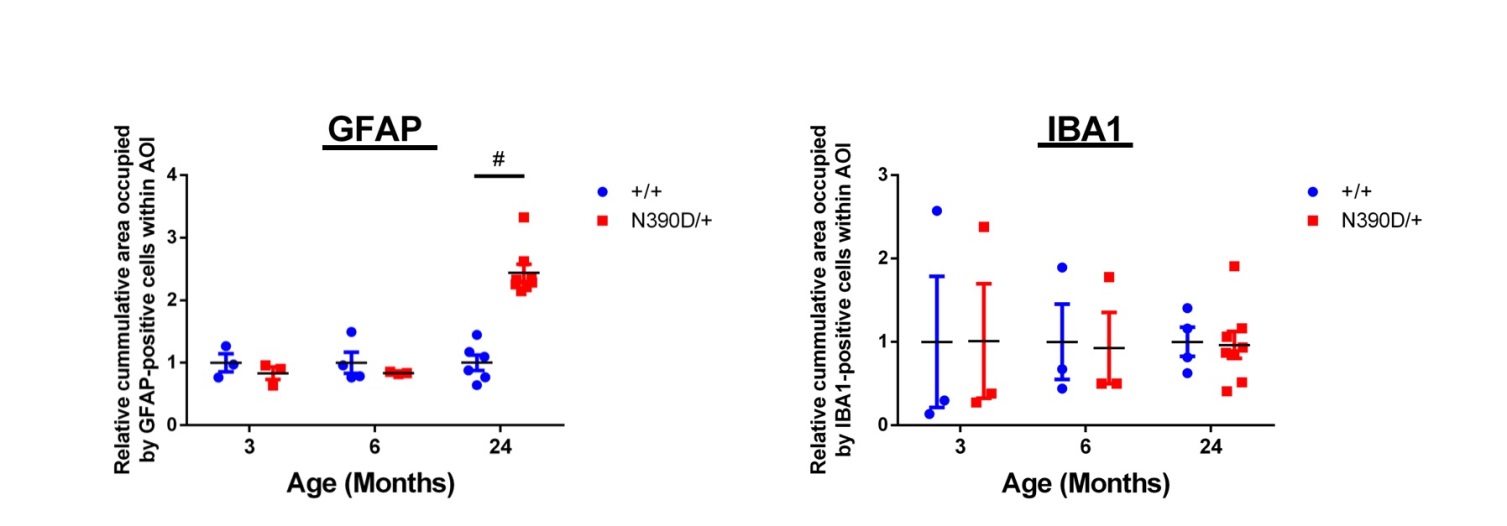
**

**Figure S5. (a)** Immunofluorescence co-staining of spinal cord sections from 3-month and 24-month old N390D/+ and +/+ male mice using anti-GFAP (green), anti-IBA1 (red) and anti-ChAT (gray). DAPI (blue) indicates the locations of the nuclei. The white line boxes mark the magnified regions from ventral horn of the spinal cord. The relative cumulative areas occupied by astrocytes **(b)** and microglia **(c)** are presented by the scatter dot plots. Only representative images of the 3-month and 24-month samples are shown. Note the increased signals of GFAP (green) in the spinal cord of 24-month old N390D/+ male mice in comparison to the age-matched +/+ male mice. N=3 (randomly chosen from each of the two independent lines) per group. The scale bars are 50 μm. #p<0.001.
