# Supplementary material for: A robust TDP-43 knock-in mouse model of ALS
Source: Acta Neuropathol Commun. 2020 Jan 21;8:3. doi: 10.1186/s40478-020-0881-5 (PMC6975031; doi:10.1186/s40478-020-0881-5)
Supplement: Supplementary file 6 — Additional file 6: Figure S6. Decreased of p62 protein level and enhanced of the proteasome activities in the spinal cord, but not the forebrain, of symptomatic N390D/+ mice. [file 40478_2020_881_MOESM6_ESM.docx]

**a**

**Spinal cord**

**
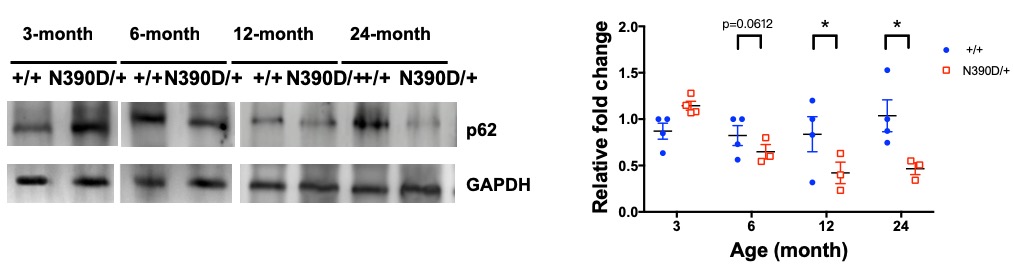
**

**b**

**
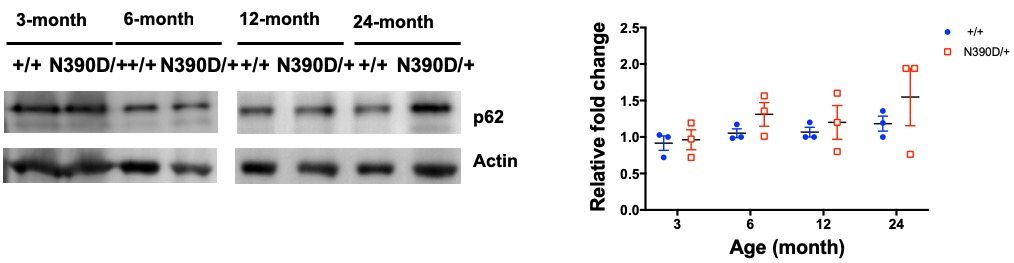
Forebrain**

**c**

**d**

**Figure S6. (a-b)** Western blotting analysis of p62 levels in the extracts of the spinal cord **(a)** and the forebrain **(b)** of +/+ and N390D/+ male mice at the ages of 3, 6, 12 and 24 months. The blotting patterns are exemplified on the left, and the statistics are shown in the scatter dot plots (mean± SD) on the right. Note the decrease of the p62 level in the spinal cord extracts, but not the forebrain extracts, from the aged (12- and 24-month old) N390D/+ mice. N=3 (randomly chosen from lines #108 and/or #361) per group. *p<0.05**. (c-d)** The proteasome activity assay of the N390D/+ (red) and +/+ (blue) spinal cord **(c)** and the forebrain **(d)** at different ages. Inhibition by MG132 was used to validate the proteasome activities. The fluorescence units were normalized against the protein concentration (mean ± SEM). Note the higher proteasome activities in the extracts of spinal cord in 6- and 24-month old N390D/+ male mice than +/+ male mice and 3-month old N390D/+ male mice. N=3 (randomly chosen from each of the two independent lines) per group. *p<0.05, **p<0.01.
