# Supplementary material for: A robust TDP-43 knock-in mouse model of ALS
Source: Acta Neuropathol Commun. 2020 Jan 21;8:3. doi: 10.1186/s40478-020-0881-5 (PMC6975031; doi:10.1186/s40478-020-0881-5)
Supplement: Supplementary file 7 — Additional file 7: Figure S7. Expression change of Bcl-2 and other TDP-43 target genes in the spinal cord of N390D/+ male mice in comparison to the +/+male mice. [file 40478_2020_881_MOESM7_ESM.docx]

**a**

**
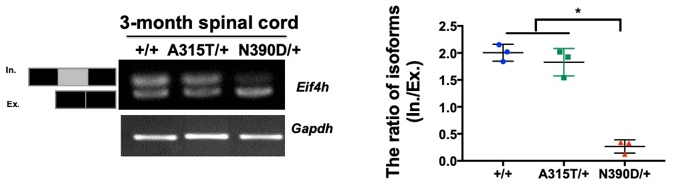
b**

**c**

**
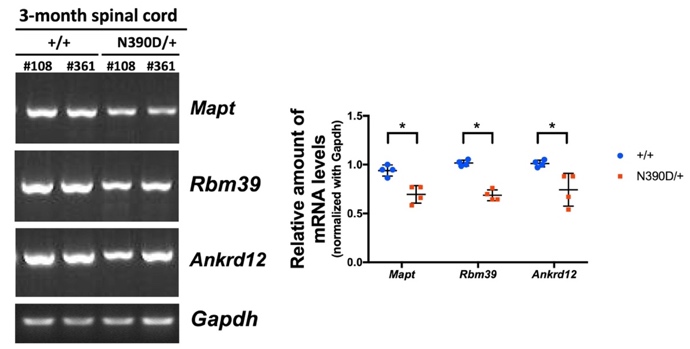
**

**Figure S7. Expression change of *Bcl-2* and other TDP-43 target genes in the spinal cord of N390D/+ male mice in comparison to the +/+male mice.**

**(a)** RT-PCR detection and statistical analysis of the levels of *Bcl-2* mRNA are shown in the left panels. Western blotting and statistical analysis of the levels of Bcl-2 protein are shown in right panels. N=3 (randomly chosen from lines #108 and/or #361) per age group. *p<0.05. **(b)** RT-PCR analysis of *eif4h* showing the alternative splicing patterns in the spinal cord of 3-month old N390D/+ and +/+ male mice, with the higher bands on gel representing the inclusive form and the lower gel bands representing the exclusive form. N=3 (randomly chosen from lines #108 and/or #361) per age group. *p<0.05. **(c)** RT-PCR data showing the decreases of the mRNA levels of the ALS-associated genes*, Mapt, Rbm39,* and *Ankrd12,* in the spinal cord of 3-month old N390D/+ male mice in comparison to the +/+male mice. N=4 (2 from #108 and 2 from #316) per group. *p<0.05.
