# Supplementary material for: A robust TDP-43 knock-in mouse model of ALS
Source: Acta Neuropathol Commun. 2020 Jan 21;8:3. doi: 10.1186/s40478-020-0881-5 (PMC6975031; doi:10.1186/s40478-020-0881-5)
Supplement: Supplementary file 8 — Additional file 8: Figure S8. Scheme of MN differentiation. [file 40478_2020_881_MOESM8_ESM.docx]

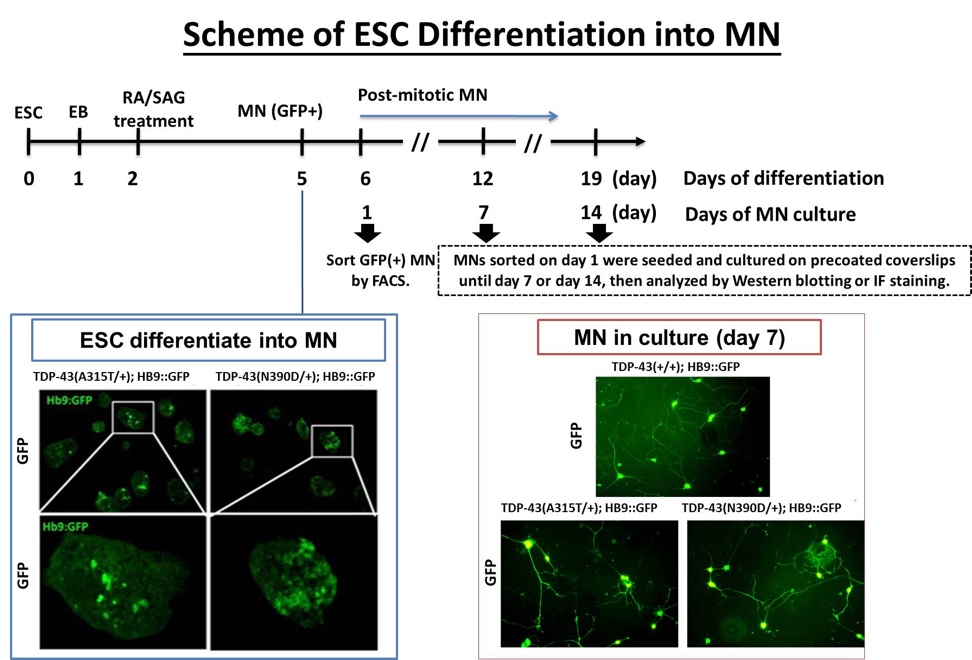


**Figure S8. Scheme of MN differentiation.** ESC from TDP-43 (+/+); Hb9:GFP, TDP-43 (A315T/+); Hb9:GFP and TDP-43 (N390D/+); Hb9:GFP mice were differentiated into spinal MN as described in Materials and Methods. Day 6 of the differentiation process would be day 1 of MN in culture when most (>65%) of the ESC were differentiated into GFP (+) MNs.
